# Integrating Protein Resistance and Bioconjugation in P(OEGMA-*co*-MAA) Brushes for Biosensing and Cell Culture: ToF-SIMS Profiling and Antibody Characterization

**DOI:** 10.1021/acsami.5c19592

**Published:** 2025-12-22

**Authors:** Katarzyna Gajos, Ostap Lishchynskyi, Paweł Da̧bczyński, Svitlana Tymetska, Łukasz Bodek, Yana Shymborska, Natalia Janiszewska, Yurij Stetsyshyn, Andrzej Budkowski

**Affiliations:** a M. Smoluchowski Institute of Physics, 37799 Jagiellonian University, Łojasiewicza 11, 30-348 Kraków, Poland; b Department of Biotechnology, Faculty of Bioscience Engineering, 26656 Ghent University, Proeftuinstraat 86, Ghent 9000, Belgium; c Jagiellonian University, Doctoral School of Exact and Natural Sciences, Łojasiewicza 11, 30-348 Kraków, Poland; d 226328 Lviv Polytechnic National University, St. George’s Square 2, Lviv 79013, Ukraine

**Keywords:** copolymer brushes, interfacial antibody state, protein immobilization, protein resistance, ToF-SIMS depth profiling

## Abstract

To provide antifouling bioactive surfaces for biosensing and cell culture, we synthesized and characterized copolymer brush interfaces with minimized nonspecific adsorption combined with adjustable high-capacity bioconjugation of functional protein and examined the interfacial protein state that determines its biological activity. Brushes were fabricated using surface-initiated atom transfer radical polymerization with silicon-grafted chains copolymerized from 2-(2-methoxyethoxy)­ethyl methacrylate (OEGMA) and methacrylic acid (MAA) taken in different proportions. For all P­(OEGMA_1–*x*_-*co*-MAA_*x*_) coatings (0 ≤ *x* ≤ 1), X-ray photoelectron spectroscopy revealed the molar fraction *x* of MAA in the brush equal to that of the reaction mixture. Time-of-flight secondary ion mass spectrometry (ToF-SIMS) showed a copolymer composition that is uniform with depth in the brush coatings, confirming a successful random copolymerization. Bioconjugation of immunoglobulin G antibody (IgG) within the brushes, enabled by the activation of MAA segments with 1-ethyl-3-(3-dimethylaminopropyl)­carbodiimide and *N*-hydroxysuccinimide (EDC/NHS), was examined together with nonspecific IgG adsorption to the nonactivated brushes using ToF-SIMS and fluorescence microscopy. Protein loading was controlled by copolymer composition and protein solution concentration. The optimal composition *x* = 0.25 was selected for the brushes with maximum bioconjugation (∼0.4 g/cm^3^) and low nonspecific adsorption. Protein loads per brush volume were estimated from ToF-SIMS depth profiles that evidenced IgG immobilization within the brush. For all P­(OEGMA_1–*x*_-*co*-MAA_*x*_) brushes with conjugated IgG antibody, the coatings with *x* = 0.25 provided the highest amount of bound antigen with an antigen binding ratio higher than that of the PMAA coatings. This observation was related to the different interfacial antibody states in both coatings (determined by the residue involvement in the coupling with the MAA segments and the dominant antibody orientation), which were investigated with multivariate principal component analysis of ToF-SIMS data. Finally, human fibroblast cell culture showed the biocompatibility of the developed copolymer brush coatings, further promoted by brush conjugation with fibronectin.

## Graphical abstract

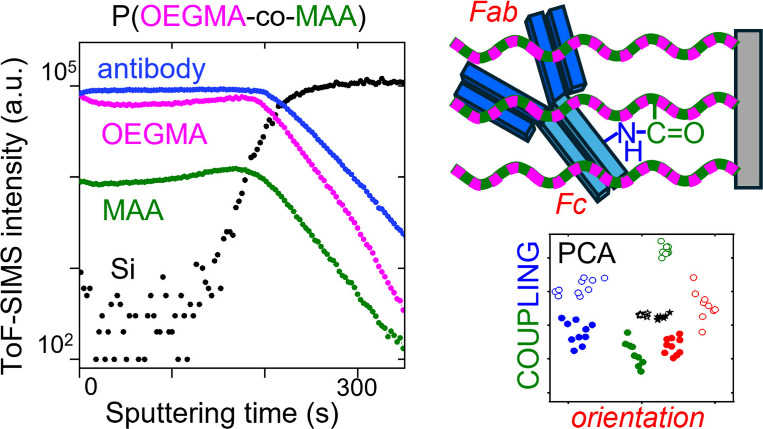

## 1 Introduction

Controlled immobilization of biomolecules on solid substrates plays a crucial role in numerous biotechnological and biomedical applications.^1,2^ Among various biomolecules, surface-immobilized functional proteins, such as enzymes, antibodies, extracellular matrix (ECM) proteins, and membrane proteins, are widely used to create bioactive surfaces for biosensing, diagnostic, purification, implant materials, cell culture, and tissue engineering.^3–5^ For optimal performance of these bioactive surfaces, the protein immobilization strategy should allow control over the amount of immobilized protein, ensure the preservation of its biological activity determined by its interfacial state (conformation and orientation),^6,7^ and minimize nonspecific adsorption.^2,8^ To meet these requirements, the functionalization of the substrate surface must be carefully designed to tailor the physicochemical properties of the material. Polymer grafting, which involves the growth of polymer chains from the initiators bound to the surface, resulting in the formation of highly stable polymer brushes, is a particularly versatile approach.^3,4^ Controlled radical polymerization methods allow tuning of chain density and brush thickness, as well as the formation of copolymers. Additionally, the possibility of postpolymerization modifications enables the introduction of a wide variety of functional groups on the substrate surface.^9^

The 3D structure of the polymer brush enables the penetration of protein molecules between polymer chains, resulting in a higher protein loading capacity compared to self-assembled monolayers (SAM) or polymer coatings.^4,5,10–12^ This type of protein adsorption within the brush is classified as ternary adsorption; other types are primary at the solid substrate and secondary at the outer edge of the brush.^13,14^ Furthermore, the amount of immobilized protein can be controlled by brush properties, such as thickness, composition and chain density, or by environmental conditions in the case of stimuli-responsive polymers.^15,16^ For biosensing applications, stable covalent immobilization of protein is preferred, which for polymer brushes can be achieved through protein-reactive side chain functional groups incorporated in the monomer or introduced during postpolymerization modification.^3–5^ The application of polymer brushes with carboxyl groups that allow the coupling of biomolecules through primary amines using EDC/NHS chemistry (based on 1-ethyl-3-(3-dimethylaminopropyl)­carbodiimide and *N*-hydroxysuccinimide) is one of the most common approaches.^4,5^ In addition, polymer brushes are advantageous for preserving the biological activity of immobilized proteins, which is a critical issue for bioactive surfaces.^4,5,10,12^ Polymer chains serve as a spacer, reducing conformational changes and steric hindrance compared to the situation of proteins directly bound to the substrate. Furthermore, the hydrophilic microenvironment that can be provided by the polymer brush, particularly that based on poly­(ethylene glycol) (PEG), poly­(oligo­(ethylene glycol)­methacrylate) (POEGMA) or poly­(2-hydroxyethyl methacrylate) (PHEMA), is essential to maintain biological activity.^3^ Additionally, these hydrophilic polymer brushes exhibit antifouling properties that prevent nonspecific adsorption, a parasitic effect in biosensing that can lead to false positive results.^3,17^ Despite the diversity of polymer brushes, ensuring all the desirable features of protein immobilization simultaneously, particularly combining antifouling properties with high-capacity covalent protein binding, remains a significant challenge. Here, copolymer brush coatings^3,4^ or mixed polymer brushes^18^ represent promising approaches. Several studies report on diblock^19–22^ or random^23,24^ copolymer brushes composed of hydrophilic polymer containing ethylene glycol methacrylate to resist nonspecific protein adsorption, and polymer with carboxylic acid^19,21,23^ or epoxide side chain^20,22,24^ for protein covalent coupling.

The comprehensive characterization of polymer brush coatings with immobilized functional proteins, including analysis of the interfacial state (involving protein conformation and orientation),^6^ should not be neglected in the development of bioactive surfaces. Among various surface-sensitive techniques, time-of-flight secondary ion mass spectrometry (ToF-SIMS) is a powerful method for the analysis of macromolecular and biomacromolecular layers.^8^ ToF-SIMS is widely applied for the analysis of the molecular composition of polymer brush coatings.^25–29^ Although ToF-SIMS offers unique capabilities for protein immobilization analysis, such as highly sensitive protein detection for antifouling evaluation or analysis of interfacial protein state, it is still rarely used for protein-functionalized polymer brushes.^6,30–32^ The development of cluster ion beam sources enables ToF-SIMS depth profiling of polymer brushes,^28,33–36^ which however has not involved an analysis of protein distribution in the brush to date.

In this work, we report the fabrication and comprehensive characterization of novel copolymer brush coatings based on 2-(2-methoxyethoxy)­ethyl methacrylate (OEGMA) and methacrylic acid (MAA), for biosensing and cell culture applications. A series of copolymer brush coatings were synthesized using atom transfer radical polymerization from a silicon substrate using OEGMA and MAA monomers in different proportions, to optimize the balance between the antifouling properties of OEGMA and the density of the protein coupling species provided by MAA. The composition of the grafted random copolymer brushes was evaluated using X-ray photoelectron spectroscopy (XPS) and ToF-SIMS and further examined through the brush thickness with ToF-SIMS depth profiling. The bioconjugation capacity of the immunoglobulin G antibody (IgG), the protein acting as a detecting molecule in immunosensors, was examined with ToF-SIMS and fluorescence microscopy after EDC/NHS activation of MAA carboxyl groups for coatings with different compositions and for various protein solution concentrations. Nonspecific adsorption was determined as well for nonactivated brushes. The P­(OEGMA-*co*-MAA) brush coatings with optimal composition, providing maximum bioconjugation and low nonspecific adsorption, were selected for further analysis. For the first time, we report ToF-SIMS depth profiling of a polymer brush with immobilized proteins to evaluate their distribution through brush thickness and estimate protein loads per brush volume. Additionally, the interfacial antibody states on P­(OEGMA-*co*-MAA) and PMAA coatings, involving the residue involvement in the coupling with MAA segments and dominant antibody orientation, were investigated with principal component analysis of ToF-SIMS data. Finally, the biological activity of the conjugated IgG antibody determined by the protein state was evaluated and the biocompatibility of the coatings was confirmed by human fibroblast cell culture.

## 2 Experimental Section

### 2.1 Fabrication of Copolymer Brush Coatings

Silicon substrates with a native SiO_2_ layer were purchased from Si-Mat (GmbH, Germany). (3-Aminopropyl) triethoxysilane (APTES, >98%), methacrylic acid (MAA, >99%), di­(ethylene glycol)­methyl ether methacrylate (OEGMA, >95%), 2-bromoisobutyryl bromide (BIBB, >98%), triethylamine (Et3N, >99.5%), sodium L-ascorbate (>98%), 2,2′-dipyridyl, CuBr_2_ (>98%) and solvents were purchased from Sigma-Aldrich (Darmstadt, Germany).

The silicon plates were washed with ethanol (>99.8%) and water and dried. The plates were placed in a vacuum desiccator or vacuum oven with a vial containing 10 drops of APTES. The chamber was then pumped down to <1 mbar, isolated from the pump, and left under vacuum for 30 s. The substrates were then annealed at 120 °C in air at atmospheric pressure for 20 min. After annealing, the substrates were reacted directly with 2-bromoisobutyryl bromide. For this, 10 mL of anhydrous tetrahydrofuran (>99.9%) was mixed with 2-bromoisobutyryl bromide (0.26 mL, 2.10 mmol) and anhydrous triethylamine (0.30 mL, 2.10 mmol) and added to an amino-functionalized substrate. Silicon plates with grafted atom transfer radical polymerization (ATRP) initiator were placed in test tubes, deoxygenated by nitrogen purging or vacuum/nitrogen cycling. Methanol (>99.8%) (16 mL), water (4 mL) and (a) OEGMA (22.6 g, 120.0 mmol) or (b) OEGMA (22.0 g, 117.0 mmol) and MAA (0.26 g, 3 mmol) or (c) OEGMA (20.3 g, 108.0 mmol) and MAA (1.0 g, 12 mmol) or (d) OEGMA (16.9 g, 90.0 mmol) and MAA (2.6 g, 30 mmol) or (e) OEGMA (11.3 g, 60.0 mmol) and MAA (5.2 g, 60 mmol) (f) MAA (10.6 g, 120.0 mmol) were mixed in a round-bottom flask sealed with a septum and deoxygenated by bubbling through nitrogen for 15 min. Then CuBr_2_ (7.4 mg, 0.033 mmol), 2,2′-dipyridyl (51.5 mg, 0.33 mmol) and sodium L-ascorbate (65.3 mg, 0.33 mmol) were added and the headspace was purged with nitrogen for 10–15 min. The mixture was stirred to dissolve the solids. Subsequently, the solution was syringed over the substrates in the deoxygenated tubes or simply poured over the substrates in a screw-top jar, which was then resealed. The samples were allowed to polymerize at room temperature. After 15 h of polymerization, the samples were removed and washed with ethanol and water.

### 2.2 Protein Immobilization

Polyclonal goat antirabbit IgG antibody (unlabeled and labeled with Alexa Fluor 488), F­(ab)_2_ domains of goat antirabbit IgG antibody, bovine serum albumin (BSA) labeled with Alexa Fluor 488, and polyclonal rabbit antimouse IgG antibody labeled with Alexa Fluor 488 were purchased from Thermo Fisher Scientific (Rockland, MA, USA). The Fc domains of the goat antirabbit IgG antibody were purchased from US Biological (Salem, MA, USA). 1-ethyl-3-(3-dimethylaminopropyl)­carbodiimide (EDC, >97%), *N*-hydroxysulfosuccinimide sodium salt (sulfo-NHS, >98%), hydroxylamine and buffers were purchased from Sigma-Aldrich (Darmstadt, Germany).

For covalent binding of antibodies to brush coatings, the carboxyl groups of the copolymer were activated with EDC/NHS by immersion in 0.04 M sulfo-NHS and 0.04 M EDC solution in 2-morpholinoethanesulfonic acid buffer (MES buffer, 0.1 M, pH = 6) for 2 h, followed by washing with distilled water and drying under nitrogen stream. Furthermore, prior to protein immobilization all substrates were soaked with phosphate buffered saline (PBS buffer, 0.15 M, pH = 7.4) for 15 min. The immobilization of goat antirabbit IgG antibody (IgG antibody) was carried out by covalent binding to EDC/NHS activated copolymer brush coatings and physical adsorption to nonactivated ones by incubation of samples with a 100 μL droplet of the 100 μg/mL solution of IgG antibody for 1 h. To examine the extent of nonspecific protein adsorption in brushes functionalized with IgG antibody, after step of immobilization of IgG antibody (application of unlabeled IgG), the coatings were incubated in 0.01 M hydroxylamine solution in PBS to hydrolyze unreacted NHS, then incubated with a 100 μL droplet of the 2 mg/mL solution of fluorescence-labeled BSA for 1 h. In turn, to examine the activity of IgG antibodies immobilized in the brush coating, after the IgG antibody immobilization step (application of unlabeled IgG), the samples were incubated with a 100 μL droplet of 10 μg/mL solution of florescence-labeled rabbit IgG as an antigen. Before examination with florescence microscopy and ToF-SIMS, all samples were extensively washed with distilled water and dried under a nitrogen stream.

### 2.3 Coatings Characterization

#### 2.3.1 Profilometry

To determine the thickness of the copolymer brush coatings, their profiles were recorded using a Dektak XT (Bruker, Bremen, Germany) profilometer equipped with a 6.5 μm radius stylus. For each sample, a scratch was made on the surface that exposed the silicon layer below, followed by the collection of six profiles across the scratch in the standard hill and valleys module. To determine the wet thickness of the polymer brush, we immersed the coatings in water overnight and dried them with a stream of nitrogen immediately prior to measurement.

#### 2.3.2 Optical Fluorescence Microscopy

Fluorescence images were collected from dried copolymer brush coatings after protein immobilization using an Olympus IX51 microscope equipped with a 100 W mercury light source. At least five images were collected for each sample using the Cell^F software, for which the average fluorescence intensity was semiquantitatively analyzed using Minkowski measurements.^37^

#### 2.3.3 X-ray Photoelectron Spectroscopy

X-ray photoelectron spectroscopy measurements were performed using a ESCALAB OXi Microscope workstation manufactured by ThermoFisher Scientific. Spectra were collected in normal emission geometry using 650 μm spot formed by a focused monochromatic Al Kα (E = 1486.6 eV) X-ray source. The flood gun was used to avoid charging effects. The measurements were taken in an ultrahigh vacuum (UHV) with a base pressure below 5 × 10^–10^ mbar. The general XPS survey spectra and high-resolution XPS spectra of the C 1s, O 1s, N 1s, and Si 2p core–shells were collected at two representative areas on each sample. Spectra were referenced to the neutral carbon C 1s peak at a binding energy of 284.80 eV.

#### 2.3.4 Time-of-Flight Secondary Ion Mass Spectrometry

The surface composition of copolymer brush coatings before and after protein immobilization was examined with ToF-SIMS using a TOF.SIMS 5 instrument (ION-TOF GmbH). Bi_3_^+^ clusters produced by a 30 keV bismuth liquid metal ion gun were used as the primary ion. For measurements in static mode, an ion dose density was lower than 10^12^ ions/cm^2^, a current was about 0.5 pA and a low-energy electron flood gun was used for charge compensation. Positive-ion high-mass resolution ToF-SIMS spectra were acquired from several nonoverlapping 200 × 200 μm^2^ areas of each sample with a resolution of 256 × 256 points. Mass calibration was carried out with H^+^, H_2_^+^, CH^+^, C_2_H_2_^+^ and C_4_H_5_^+^ peaks, with mass resolution (m/Δm) at C_4_H_5_^+^ greater than 8000 for all spectra. Multivariate analysis of TOF-SIMS data was performed with PCA using the PLS Toolbox (eigenvector Research, Manson, WA) for MATLAB (MathWorks, Inc., Natick, MA). Before PCA was run, the intensities of selected peaks from each spectrum were normalized to total ion intensity and mean-centered. Additionally, ToF-SIMS depth profiling was performed for bare P­(OEGMA-*co*-MAA) 75/25 copolymer brush coating, as well as for EDC/NHS activated coating after IgG antibody coupling and nonactivated coating after IgG antibody adsorption. The depth profiles of the samples were obtained in the dual beam mode. The 2.5 keV argon gas cluster ion beam (Ar-GCIB) was used to sputter a 350 × 350 μm^2^ area, and the Bi_3_^+^ ion beam was used to analyze the 150 × 150 μm^2^ area concentric to the sputtered area. For each sample, three depth profiles were recorded for negative and positive ions from nonoverlapping areas.

### 2.4 Cell Culture, MTT, and Immunofluorescence Assays

#### 2.4.1 Cell Culture

Human primary dermal fibroblasts neonatal (HDFn; ATCC, PCS-201–010) were cultured in Dulbecco’s modified eagle medium (DMEM) with high glucose (Sigma-Aldrich, D6429), which was supplemented with 10% fetal bovine serum (Sigma-Aldrich, F9665) and 1% penicillin–streptomycin–neomycin solution (Sigma-Aldrich, P4083) in culture flasks, at 37 °C in a humidified atmosphere in CO_2_ incubator providing 95% air and 5% CO_2_. Glass (or silica) coverslips 15 × 15 (or 10 × 10) mm^2^ coated with copolymer brushes were placed at the bottom of the cell culture plate (12-well; flat bottom). The samples were sterilized with 96% ethanol for 5 min, then rinsed twice with sterile, distilled water and left in a sterile PBS buffer for 2 h under a laminar flow chamber (Nu425, NuAire). A part of the samples, activated earlier with EDC/NHS, was then functionalized with human fibronectin (Invitrogen, 43130) applying a 200 μg/mL solution and an incubation time of 1 h. After that, HDFn fibroblasts were seeded on coatings at a concentration of 5000 cells/cm^2^. Cell culture plates were then incubated in the CO_2_ incubator for 1, 3, or 5 days. The medium was replaced after 24 and 96 h of the study. For each experimental sequence, three identical samples were prepared and measured. All experiments were repeated at least three times in a time frame to prove the reproducibility of the results.

#### 2.4.2 MTT Assay

Cell viability was verified using a microculture tetrazolium colorimetric test (MTT, Cell Proliferation Kit I, Sigma-Aldrich, 11465007001). Briefly, fibroblasts were cultured on silica coverslips coated with copolymer brushes in a multiwell plate (12 wells) in 1 mL of the corresponding culture medium. On the day of the experiment, the coverslip with polymer was transferred with 1 mL of medium to a new 12-well plate to avoid studying the response of cells that did not grow directly on the polymer. Next, 100 μL of MTT reagent (tetrazolium salt) was added to the cells in the culture medium. Cells were incubated at 37 °C in the incubator for 4 h. Then 1 mL of the solubilization buffer was added to each well. The plate was left overnight in the incubator in a humidified atmosphere at 37 °C and 5% CO_2_. The MTT method is based on the reduction of the tetrazolium compound by viable cells to generate a colored formazan product that is soluble in a cell culture medium. The resulting colored solution was quantified by a scanning multiwell spectrophotometer (SPECTROstar Nano, BMG Labtech). The final volume of 2.1 mL was pipetted onto a 24-well plate with 600 μL per hole. The absorbance was determined in the 24-well for each time frame of 1, 3, or 5 days at OD = 560 nm. The MTT assay was repeated at least three times for each time point.

#### 2.4.3 Immunofluorescence Assay

For fluorescent staining of actin and cell nuclei, the following protocol was applied. First, cells were fixed on the substrate by immersion in a 3.7% paraformaldehyde solution in PBS (Thermo Scientific, 169650010) for 15 min at 37 °C. Subsequently, cells were permeabilized with 0.1% Triton X-100 solution (Sigma, T8787) at room temperature for 8 min, samples were washed with PBS buffer for 2 min. To dye the actin cytoskeleton and cell nuclei, the samples were incubated with a solution of Alexa Fluor 488 conjugated with phalloidin (Alexa Fluor 488 Phalloidin, Thermo Fisher Scientific, A12379) in 400× dilution, a 2 μg/mL solution of Hoechst 34580 dye (Thermo Fisher Scientific, H21486) for 60 min. The cells were then thoroughly washed 2 times for 5 min with PBS buffer and 5 min with water. Finally, stained samples were mounted on glass slides in DePex medium (Serva) and stored at 18 °C. Fluorescence images were collected using the Olympus IX51 microscope equipped with a 100 W Mercury light source (Olympus U-LH100HG), a U-MWIG2 filter, and one of U-MNB2. For image processing, ImageJ FIJI was used. For each experimental run, 5 fluorescent images from three substrates with stained cells were collected.

## 3 Results and Discussion

The P­(OEGMA-*co*-MAA) copolymer brush coatings were fabricated using the SI-ATRP method, consisting of silicon surface functionalization with amino-terminated APTES film, grafting of the ATRP initiator, and finally brush polymerization using different ratios n/m of OEGMA and MAA monomers in the reaction mixture (Scheme S1, Supporting Information, Table 1). Compared to surface-initiated zerovalent metal-mediated controlled radical polymerization (SI-Mt0CRP), the SI-ATRP strategy offers several distinct advantages in terms of polymerization efficiency, monomer consumption, chain length controllability, and functional versatility. SI-ATRP allows precise control over the growth of polymer chains, resulting in narrow molecular weight distributions and well-defined grafting densities.^38^ This high level of control reduces monomer waste and enables the preparation of polymer brushes with predictable thickness and architecture. While SI-ATRP can be sensitive to oxygen, recent adaptations such as sacrificial initiators or oxygen-tolerant ATRP protocols allow polymerization under ambient conditions without significant loss of efficiency.^39^ In addition, SI-ATRP is compatible with a wide range of monomers, including functionalized monomers that may not be suitable for other polymerization techniques. Therefore, for the preparation of well-defined polymer brushes with reproducible properties, SI-ATRP provides clear advantages over SI-Mt0CRP.

**Table 1:** Composition of OEGMA/MAA Reaction Mixtures and Determined Parameters of Synthesized P­(OEGMA-*co*-MAA) Brush Coatings: XPS Mole Fraction (*x*) of MAA, Dry Brush Thickness (*h*_dry_), Thickness Ratio of Brush in the Swollen and Dry State (*h*_wet_/*h*_dry_), and the Resultant (See Table S3 for Details) Areal Number Density of Brush Chains (σ) and Chain Segments (Total, OEGMA, MAA)

OEGMA/MAA ratio (n/m) in reaction mixture	*x*, MAA mole fr.	*h*_dry_, nm	*h*_wet_/*h*_dry_	σ, chains/nm^2^	mers/nm^2^	OEGMA mers/nm^2^	MAA mers/nm^2^
100/0	0	53.9	1.6	0.28	186	186	0
97.5/2.5	0.025	52.5	1.6	0.27	185	180	5
90/10	0.1	57.4	1.4	0.33	211	190	21
75/25	0.25	54.6	1.5	0.33	224	168	56
50/50	0.5	35.4	1.8	0.28	177	89	88
0/100	1	24.7	2.2	0.29	223	0	223

Before applications of proteins (Subsections [Sec sec3.2] and 3.3) or cells (Subsection [Sec sec3.4]), the synthesized P­(OEGMA-*co*-MAA) copolymer brush coatings were subjected to a multistep washing procedure that included repeated rinsing with deionized water and ethanol to remove any loosely bound metal species. To verify the possible presence of copper residues, all samples were analyzed by ToF-SIMS. No copper signal above the detection limit (evaluated to be below 1 ppm) was found for any of the coatings, except for the sample coated with PMAA (see Figure S1 in Supporting Information). The weak signal observed in this case can be attributed to the complexation of trace Cu^2+^ ions with carboxylic groups of PMAA, which are known to exhibit a high affinity for divalent metal cations. Our previous studies^40,41^ have shown that polymer coatings initially containing copper can be fully biocompatible. In line with these results, the present analysis confirms that the polymer surfaces investigated here contain no detectable copper residues, except for trace ionic forms on the PMAA coating, which are not expected to influence biological performance. In addition, only copper concentrations in polymer brush decades higher were found to affect protein adsorption.^40^

### 3.1 Characterization of the P­(OEGMA-*co*-MAA) Copolymer Brush Coatings with Different Composition

The molecular composition of P­(OEGMA-*co*-MAA) n/m copolymer brushes was determined using XPS and ToF-SIMS techniques, which provided chemical bonding and molecular information, respectively.

#### 3.1.1 Chemical Bonding Information

XPS data were collected for the surfaces functionalized with APTES with the grafted ATRP initiator before and after polymerization of the P­(OEGMA-*co*-MAA) brushes (Figure 1). Organic coatings are characterized by photoelectrons emitted by the elements such as nitrogen (N 1s) from APTES, bromine (Br 3d) from the ATRP initiator and growing polymer chains, oxygen (O 1s) and carbon (C 1s) specific for all samples. In particular, the C 1s core-level spectra (Figure 1a–d) can be resolved into four contributions, corresponding to unfunctionalized (hydro)carbon C–C (green line, 284.8 eV), carbon C–N,O with C–N and C–O bonds (violet line, 286.3 eV), carbon (N−)C=O with N–C=O and C=O environments (cyan line, 287.9 eV), and carbon with O–C=O bonds (orange line, 288.6 eV). To determine the molecular composition of P­(OEGMA-*co*-MAA) brushes, the XPS atomic concentrations of carbon in different environments should be corrected for contributions from the ATRP initiator and adventitious carbon (aC).^42^ Both contributions are revealed by the XPS data recorded for the surfaces with grafted ATRP initiator. First, the ratio of Br and N signals reveals the mole fraction of APTES molecules functionalized with the initiator, *y* = 0.69(9). Second, the intrinsic composition of the organic layer with the ATRP initiator is defined by the chemical structure (Figure 1a) to produce the contribution to the XPS atomic concentration of (N−)C=O carbons from amide bonds, proportional to *y* and specified by the measured value, the contributions of C–C and C–N,O proportional to (2 + 3*y*) and 1, respectively, without the contribution of O–C=O (see Table S1). Third, these contributions are subtracted from the measured values to characterize adventitious carbon. Finally, to correct the XPS atomic concentrations of carbon in the polymer brush samples, we assumed that the contributions due to adventitious carbon are the same as determined for the ATRP initiator samples, but the contributions due to the ATRP initiator are proportional to the nitrogen concentration normalized by its value for the samples of the ATRP initiator alone (Table S1). The relative intensities of the peaks, reflecting different carbon bonding environments, in the C 1s core-level spectra of the copolymer brushes are presented in Figure 2a: The original (measured) and corrected (intrinsic) values are marked with open and solid symbols, respectively. To determine the mole fraction *x* of MAA in the copolymer brushes (Figure 2b), we used the intrinsic relative intensity of the peak corresponding to theC–N,O environment in the C 1s core level spectra (Figure 2a), which follows the formula (5 – 5*x*)/(9 – 5*x*). The XPS results of Figure 2b not only verify the successful fabrication of P­(OEGMA-*co*-MAA) copolymer brush coatings, but also show a linear relationship between the mole fraction *x* of MAA in the copolymer brush and in the reaction mixture, both varied between 0 and 1. Furthermore, the equality concluded here between the molar composition of MAA in the copolymer brush and that in the reaction mixture is manifested by the resulting predictions for the relative peak intensities reflecting different carbon environments in the C 1s core level spectra (lines in Figure 2a), consistent with the corrected experimental values (solid symbols). This suggests, in line with the Mayo–Lewis theory, ideal *random* copolymerization. The mer distribution along the copolymer chain is controlled by the reactivity ratios of both monomers in the reaction mixture (r_MAA_ and r_OEGMA_), which also determine the relation between the composition of the copolymer chain and the reaction mixture. The copolymerization of MAA and poly­(ethylene glycol)­monomethacrylate PEGMA, with a chemical structure similar to that of OEGMA, was previously reported with the ratios r_MAA_ = 1.03 and r_PEGMA_ = 1.02.^43^ Using these values as representative estimates results in the predicted molar compositions of the P­(OEGMA-*co*-MAA) copolymer brushes equal to those determined with XPS (see Table S2).

**Figure 1:**
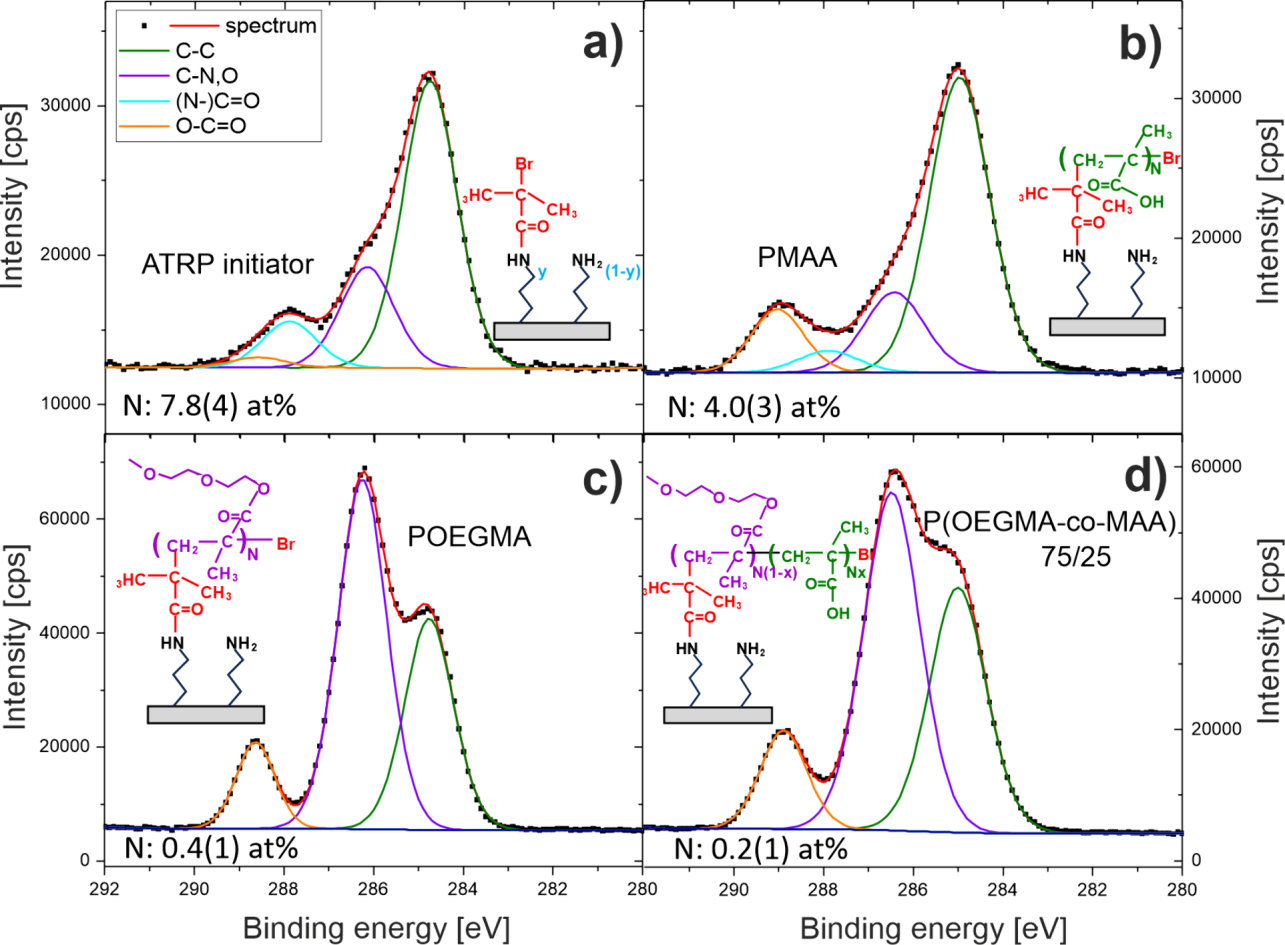
XPS C 1s core-level spectra of APTES-functionalized silicon with the grafted ATRP initiator, (a) before and after the fabrication of (b) PMAA, (c) POEGMA, and (d) P­(OEGMA-*co*-MAA) 72/25 brush coatings.

**Figure 2:**
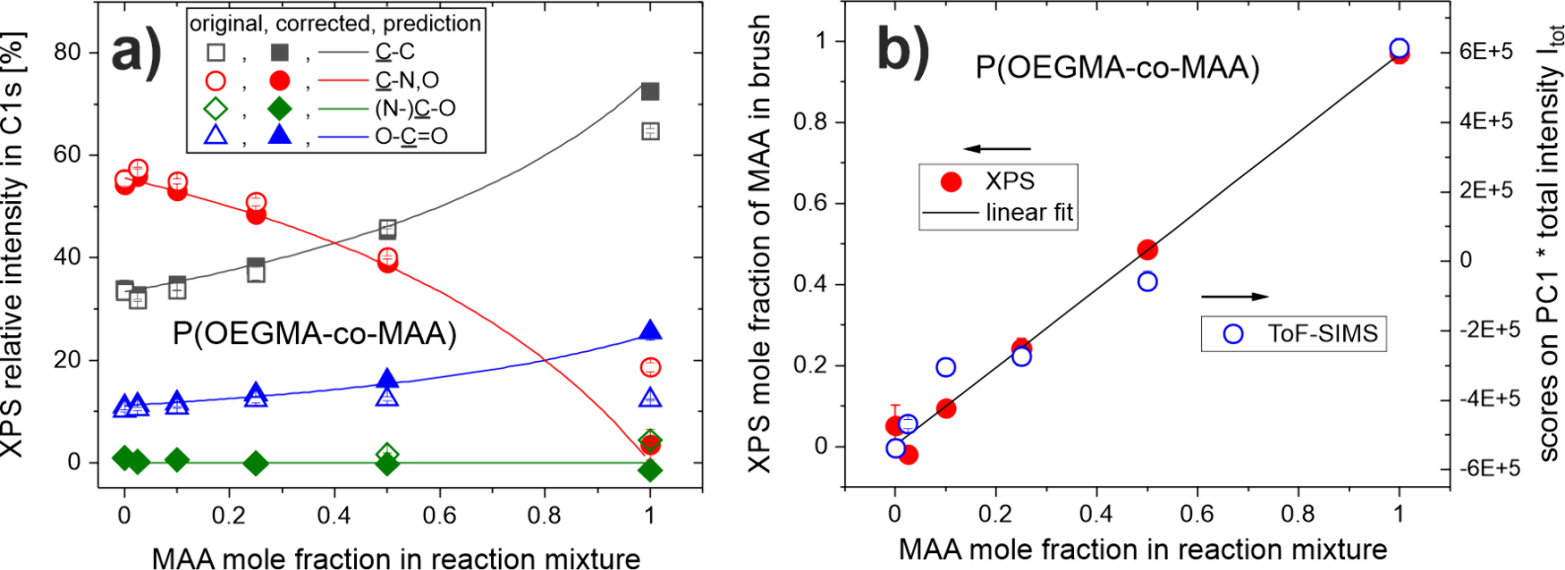
(a) Relative intensities of the peaks corresponding to the C–C (black), C–N,O (red), (N−)C=O (green) and O–C=O bonds (blue) in the XPS C 1s core-level spectra of P­(OEGMA-*co*-MAA) brush coatings. The original measured values (open symbols) and the values corrected for the ATRP initiator and adventitious carbon (solid symbols) are presented together with the predictions (lines) from the linear fit to the XPS data shown in (b). (b) Relationship between the molar MAA fraction in the reaction mixture and in the copolymer brush determined with XPS (solid red circles) and ToF-SIMS augmented with PCA (open blue circles). The ToF-SIMS data reflect the PC1 scores (see Figure 3b) multiplied by the total ion intensity *I*_*tot*_.

#### 3.1.2 Molecular Characterization and Depth Profiles of the Copolymer Brush

To corroborate the XPS conclusions on the basis of chemical bonding information on the molar composition and mer distribution of copolymer brush chains, ToF-SIMS was applied to provide molecular characterization and depth profiles of the copolymer brush. ToF-SIMS working in static mode conditions validates the successful fabrication of P­(OEGMA-*co*-MAA) copolymer brush coatings. This is illustrated by the representative ToF-SIMS positive ion spectra (Figure 3a), recorded from the brushes polymerized from OEGMA and MAA monomers, as well as their 75/25 mixture. The spectrum of the P­(OEGMA-*co*-MAA) copolymer brush reveals two series of signals, characteristic for the OEGMA and MAA segments, respectively, with hydrocarbon ions containing oxygen (marked in blue), such as CHO^+^ (*m*/*z* = 29), C_2_H_5_O^+^ (*m*/*z* = 45), C_3_H_7_O^+^ (*m*/*z* = 59), C_6_H_9_O_2_^+^ (*m*/*z* = 113),^28,32^ and hydrocarbon ions without oxygen (marked in red), such as C_3_H_5_^+^ (*m*/*z* = 41), C_3_H_7_^+^ (*m*/*z* = 43), C_4_H_7_^+^ (*m*/*z* = 55), C_5_H_9_^+^ (*m*/*z* = 69). To examine variations in molecular composition between polymer brushes, 60 ToF-SIMS measurements of the P­(OEGMA-*co*-MAA) n/m copolymer brushes with different compositions (listed in Figure 3b) were simultaneously examined with respect to the normalized intensities of 24 signals, originating from the OEGMA and MAA segments (listed in Figure 3c), using a multivariate principal component analysis (PCA). Some oversaturated signals that stand out in the ToF-SIMS spectra were excluded from the PCA analysis. The first principal component (PC1), which represents the direction of the major uncorrelated variation within the data set, captures most of the total variance (88.56%). The relationship of PC1 with the original mass signals (Figure 3c) shows negative and positive loadings originating from the ion fragments characteristic for MAA and OEGMA, respectively. Therefore, PC1 should distinguish between the molecular composition of the copolymer brush rich in MAA and OEGMA. In fact, PC1 scores (Figure 3b) are spanned by homopolymer brush data, with negative values for PMAA and positive values for POEGMA. In turn, the data of the copolymer brushes show a decrease in PC1 scores, reflecting a higher MAA brush concentration for a higher MAA content in the reaction mixture. However, the relationship between PC1 scores and monomer composition during polymerization is not truly monotonic (Figure 3b) when homopolymer and copolymer brush data are combined, especially for the P­(OEGMA-*co*-MAA) 97.5/2.5 copolymer. This reflects a slight nonlinearity between copolymer composition and scores, because the latter originate from the normalized ToF-SIMS intensities applied in PCA.^28^ In the absence of matrix effects on ion formation, the linearity with surface composition is expected not only for the absolute ToF-SIMS intensities but also for the total ion intensity *I*_*tot*_ used for their normalization. Therefore, PC1 scores vary with the composition following a relation described as the ratio of two linear expressions. Consequently, the local surface composition is better expressed by the PC1 scores multiplied by the total ion intensity *I*_*tot*_ than by the PC1 scores alone.^28^ The first quantity is plotted in Figure 2b to show the monotonic relation between the molar fraction of MAA in the reaction mixture and the copolymer brush determined with ToF-SIMS augmented with PCA (open blue circles), mimicking the linear relation determined with XPS (solid red circles and linear fit). Overall agreement between the ToF-SIMS and XPS results presented in Figure 2b confirms the assumptions taken to correct the XPS data for the adventitious carbon and ATRP initiator. In turn, small discrepancies for some copolymer brushes can be related with the molar composition of their outermost nanometer region, sampled with ToF-SIMS, apparently slightly different from that sampled with XPS within a 10 nm subsurface region.

**Figure 3:**
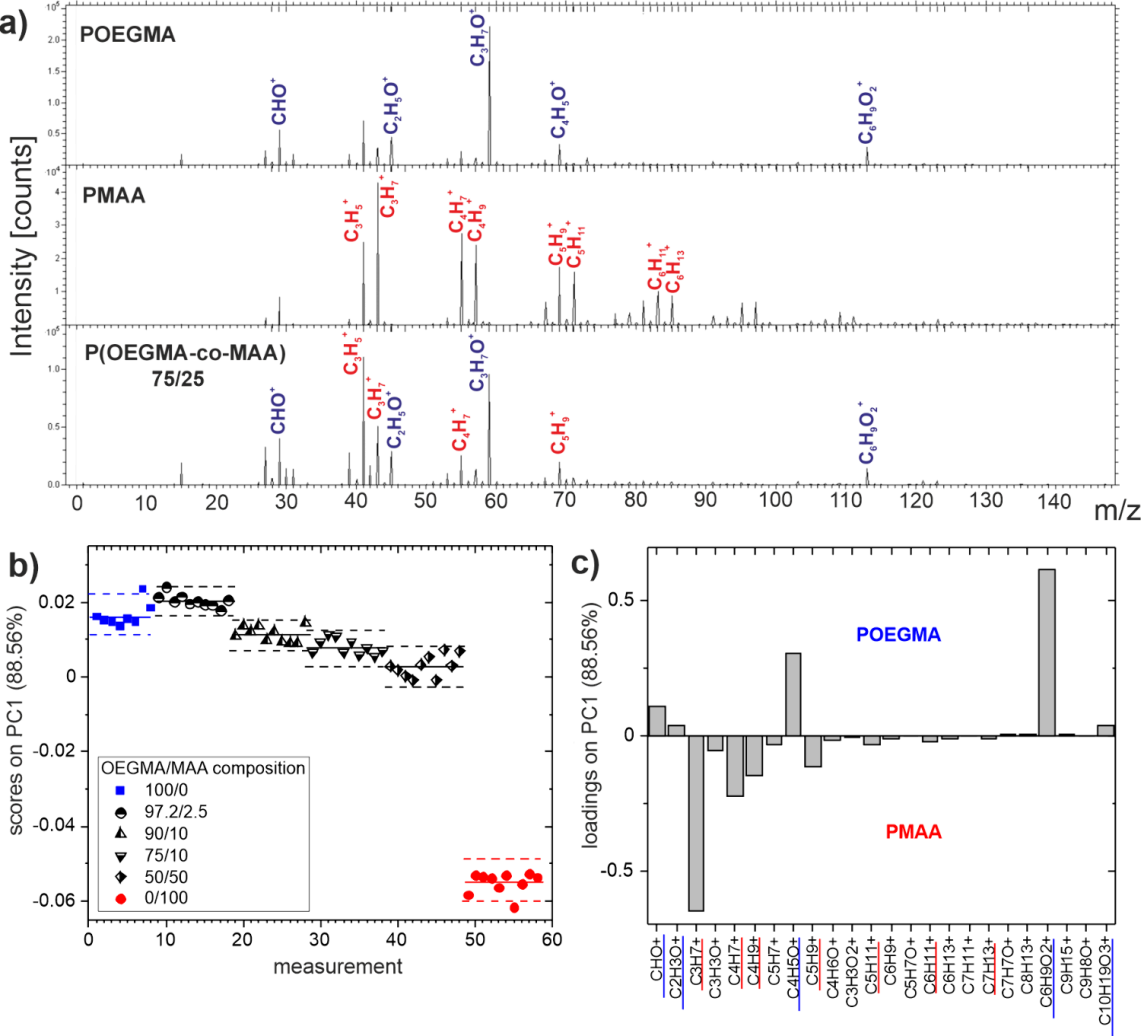
(a) ToF-SIMS positive ion spectra recorded for POEGMA, PMAA, and P­(OEGMA-*co*-MAA) 72/25 brush coatings. The ions characteristic for the OEGMA and MAA segments are marked in blue and red, respectively. (b) PCA scores plot of homopolymer, POEGMA and PMAA, and copolymer P­(OEGMA-*co*-MAA) brush coatings (cf. ToF-SIMS data in Figure 2b). The dashed lines represent the 95% confidence limits for each group of data points. (c) Corresponding loading plot that relates PC1 with ToF-SIMS signals.

To examine the concentration of OEGMA and MAA mers through brush depth, the dual beam profiling mode of ToF-SIMS was applied. P­(OEGMA-*co*-MAA) 75/25 copolymer brush coating was sputtered using an argon gas cluster ion gun (Ar_1000_^+^), which is a powerful sputtering source for depth profiling of organic materials, as it reduces polymer cross-linking and preserves molecular information during erosion compared to other sources.^44,45^ The ToF-SIMS depth profiles are provided by the measured secondary ion intensities of characteristic ions plotted as a function of the sputtering time (Figure 4a). The intensities of the ions characteristic of particular mers, C_4_H_5_O_2_^–^ (*m*/*z* = 85) for OEGMA and C_4_H^–^ (*m*/*z* = 49) for MAA, reflect their volume fractions (discussed in detail in Section 3.2.1) and remain constant during sputtering. This confirms the fabrication of a *random* copolymer brush with a uniform composition through the brush down to the silicon surface. An increase in the intensity of the CNO^–^ ions (*m*/*z* = 42), characteristic of the APTES amide links with the ATRP initiator (APTES-ATRP), along with the growth of the Si^–^ signal and the decrease in the copolymer signals, reflects the interfacial region of the grafting surface (Figure 5a).

**Figure 4:**
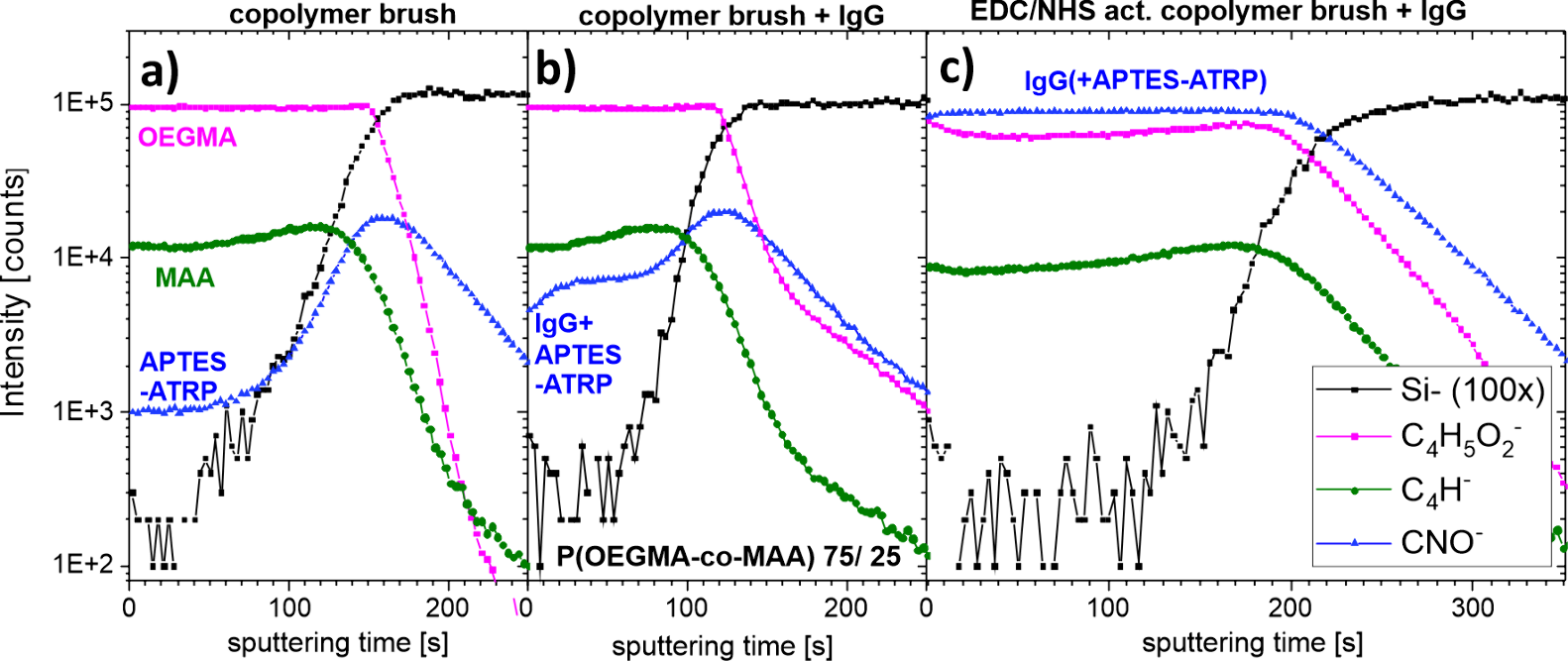
ToF-SIMS depth profiles of P­(OEGMA-*co*-MAA) 72/25 brush coatings after synthesis (a), followed by physical adsorption (b) or covalent immobilization of the IgG antibody (c), the latter enabled by an earlier activation of the brush coatings using the EDC/NHS covalent coupling procedure. The negative ions plotted in (a)–(c) are characteristic for the silicon substrate (Si^–^), OEGMA (C_4_H_5_O_2_^–^) and MAA (C_4_H^–^) segments of the copolymer brush, the ATRP initiator bound to APTES (CNO^–^) and the IgG protein (also CNO^–^).

**Figure 5:**
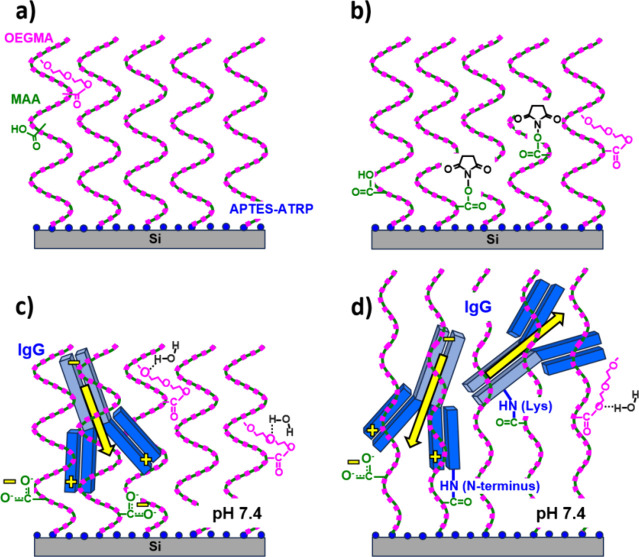
Schematic presentation of P­(OEGMA-*co*-MAA) copolymer brush coatings after synthesis (a), followed by physical adsorption (c) or covalent immobilization of the IgG antibody (d), the latter enabled by an earlier activation of the brush coatings using the EDC/NHS covalent coupling procedure (b). IgG adsorption and bioconjugation are opposed by hydration shells formed around OEGMA segments by H-bonds with water molecules (c, d). They are favored by electrostatic interactions between antibody dipoles and negatively charged MAA segments (c) or by conjugation of antibody amines with the EDC/NHS activated MAA segments (d). The IgG orientation results from competing electrostatic interactions (dipole-dipole and dipole-MAA segments) and conjugation of amines with different locations on the antibody (all vs F­(ab)_2_ domains).

#### 3.1.3 Graft Density, Morphology, and Wettability

Brush coatings made of P­(OEGMA_1–*x*_-*co*-MAA_*x*_) with different composition (the mole fraction *x* of MAA) were characterized by profilometry, and their thickness in the dry and swollen states (Table 1) was determined before and after immersion in deionized water, respectively.^41,46^ The thickness of the coatings (in the dry state, *h*_dry_) is approximately 55 nm for *x* not greater than 0.25, and noticeably thinner for MAA-rich coatings, with *h*_dry_ ∼ 35 nm and ∼25 nm for *x* = 0.5 and 1.0, respectively. Based on the thickness ratio (*h*_wet_/*h*_dry_) of the brush in the swollen state (*h*_wet_ = C_w_·N·σ^1/3^) and in the dry state (*h*_dry_ = C_d_·N·σ), the areal number density of the brush chains (σ) was estimated as previously reported^41,46^ for the coefficients (C_w_, C_d_) presented in Table S3. The resulting brush grafting densities are shown in Table 1, together with the areal number densities of the total, OEGMA and MAA chain segments, calculated (see Table S3) for the brush composition provided by XPS. The estimated (areal number) densities of the brush and (total) chain segments vary only slightly (<12%) with the brush composition, with values centered around their means of 0.30 chains/nm^2^ and 201 mers/nm^2^, respectively. These density values of the brush grafting are comparable to those reported for POEGMA brushes (0.2–0.3 chains/nm^2^)^47^ and PMAA brushes (0.2–0.4 chains/nm^2^)^41^ grafted from silicon or glass substrates using SI-ATRP. The density of the MAA segments increases to 223 mers/nm^2^, and that of the OEGMA segments decreases starting from 186 mers/nm^2^, with increasing MAA mole fraction in the brush (Table 1). Similar number density values of chain segments (∼198 mers/nm^2^) were obtained for the PMAA brushes synthesized from MAA, as here.^41^ The estimated brush grafting density (σ ∼ 0.30 chains/nm^2^) is the result of the surface density of the APTES molecules (up to 4.0 APTES/nm^2^,^48^), their functionalization with the ATRP initiator (with a rate of 0.69, see Section 3.1.1), and the successful growth of chains from the APTRP initiator sites during SI-ATRP polymerization (with initiation efficiency ∼ 10% ^49^).

In the next step, the morphology of the coatings with different compositions was examined with AFM (for experimental details see Section S1 in Supporting Information). Topography AFM images recorded in the dry state at room temperature are presented in Figure S2. The PMAA homopolymer brush coatings have an island-like structure with roughness described by a root-mean-square (RMS) value ∼2.5 nm. The morphology of POEGMA homopolymer brush coatings is homogeneous with an RMS value ∼1.8 nm. In turn, all copolymer P­(OEGMA-*co*-MAA) brush coatings are homogeneous and relatively smooth, with an RMS value of less than 1 nm, regardless of the MAA molar fraction. Additionally, optical microscopy and ToF-SIMS maps of characteristic ions (Figure S3) confirmed the macroscale uniformity and continuous coverage of the grafted P­(OEGMA-*co*-MAA) brush surfaces, in agreement with AFM and profilometry results.

Finally, the wettability of the coatings was determined with measurements of water contact angle (CA) performed at room temperature for the ‘as prepared’ coatings (Table S4 and Figure S4, for experimental details see Section S1 in Supporting Information). The PMAA homopolymer brush coatings are characterized by CA ∼ 53 deg. This reflects the application of methacrylic acid (MAA) for brush polymerization in this work, resulting in more hydrophobic surfaces than those of PMAA brushes polymerized from sodium methacrylate (CA ∼ 35 deg).^41,50^ Higher CA values (∼60 deg at room temperature) were reported for PMAA brush coatings synthesized from MAA with a lower grafting density (0.2 chains per nm^2^).^41^ In turn, the CA value determined for POEGMA homopolymer brush coatings ∼66.5 deg is in agreement with the values determined under the same conditions for POEGMA brush coatings that respond to temperature and pH.^28,51^ The hydrophobicity of the P­(OEGMA-*co*-MAA) copolymer brush coatings decreases with the MAA molar fraction *x*, to reach for *x* = 0.5 the CA value of the PMAA homopolymer brush coating.

### 3.2 Antibody Bioconjugation and Physical Adsorption to P­(OEGMA-*co*-MAA) Brush Coatings

The desirable composition of the P­(OEGMA-*co*-MAA) copolymer should provide an optimal balance between the carboxylic MAA units, allowing covalent bioconjugation, and the hydrophilic OEGMA units responsible for protein resistance. XPS and ToF-SIMS results confirmed a successful random copolymerization, while ToF-SIMS profiling demonstrated a uniform copolymer composition through the brush (for the 75/25 composition). Here, we first extend the ToF-SIMS profiling analysis of this copolymer brush to evidence protein conjugation and protein resistance. Then, we apply florescence microscopy and ToF-SIMS (static mode) data to systematically evaluate the protein coupling and low-fouling properties of the P­(OEGMA-*co*-MAA) brushes with different copolymer compositions. The best compromise between bioconjugation and protein resistance is obtained for the 75/25 composition (chosen for ToF-SIMS profiling).

#### 3.2.1 ToF-SIMS Depth Profiles of Proteins within the Copolymer Brush

Protein concentration profiles as a function of depth within polymer brushes can provide direct evidence for different types of adsorption, classified as primary at the solid substrate, secondary at the outer edge of the brush, and ternary within the brush.^13,14^ So far, such profiles have been provided for proteins within *homopolymer* brush chains by neutron reflectometry^13,14^ or XPS combined with cluster ion sputtering,^52^ with system components resolved by hydrogen/deuterium contrast^13,14^ or elemental information,^52^ respectively. Here, dual beam ToF-SIMS profiling was applied, combining *superior molecular specificity* with cluster ion sputtering to examine the spatial distribution of the IgG antibody within the *copolymer* brush P­(OEGMA-*co*-MAA) 75/25. The situation after brush synthesis (Figure 4a, discussed in Section 3.1.2) is compared with that after physical adsorption (Figure 4b) or chemisorption of the IgG protein (Figure 4c), the latter enabled by earlier brush activation using the EDC/NHS covalent coupling procedure (Figure 5a,b, see the next Section). The intensities of the characteristic negative ions plotted against the sputter time (Figure 4) reflect the volume fractions^13,14,53^ of the OEGMA segments (C_4_H_5_O_2_^–^), the MAA segments of the copolymer brush (C_4_H^–^), and jointly (CNO^–^) the IgG protein and the ATRP initiator bound to APTES (hereinafter referred to as APTES-ATRP) as a function of depth within the brush. The signals of the copolymer segments remain nearly constant during sputtering, also for protein-containing samples, but their intensities are visibly reduced for the brushes with bioconjugated IgG (Figure 4c). In turn, the CNO^–^ signal for the samples with proteins, compared to that of the brush ‘as prepared’, indicates *ternary protein adsorption* (within the brush), which is weak for the physically adsorbed IgG antibody (Figure 4b) and very high for the chemisorbed IgG antibody (Figure 4c). To quantitatively examine these features, the average intensities of the characteristic ions along the brush depth (with thickness specified by the half-maximum of the Si^–^ signal) are presented in Table 2. The corresponding average volume fractions of the brush components (APTES-ATRP, MAA, OEGMA) are juxtaposed in Table 2, calculated based on XPS data for the brush ‘as prepared’, and then rescaled to the samples with proteins, using the average ToF-SIMS intensity of the C_4_H^–^ and C_4_H_5_O_2_^–^ ions. In turn, the volume fractions of the brush components provide, after subtracting from unity, those of the IgG protein. The latter can also be related between the physically adsorbed and the chemisorbed protein, using the relative net intensity of the CNO^–^ ions obtained by subtracting the value of the ‘as-prepared’ coating. The values presented in Table 2 show that the average volume fractions of the brush components are reduced by 1.02 and 1.40 times for the physically adsorbed and conjugated IgG protein, to introduce the protein with a total mass loaded per brush volume Γ/*h* of 0.03 and 0.39 g/cm^3^, respectively. For the brush thickness of 54.6 nm, these values correspond to the total protein mass per surface area Γ of 1.4 and 21.5 mg/m^2^, respectively, marked as blue triangles in Figure 6a (discussed in the next Section). As a result of volume conservation, a related increase in brush thickness is expected due to protein loading, and this is reflected in the extended total sputtering time (Figure 4c).

**Table 2:** Analysis of ToF-SIMS Depth Profiles of P­(OEGMA-co-MAA) 72/25 Brush Coatings before and after Physical Adsorption or EDC/NHS Coupling of the IgG Antibody (Figure 4)

	Average ion intensity along the brush depth^a^			Average volume fractions (%)					
P(OEGMA-*co*-MAA) 72/25 brush coatings	CNO^–^	C_4_H^–^	C_4_H_5_O_2_^–^	amino acid	APTES-ATRP	MAA	OEGMA	Total IgG mass per brush volume Γ/*h*, g/cm^3^	Relative brush thickness change^f^
as prepared	1507	13126	94284	0	2.4^b^	11.1^b^	86.5^b^	0	1
after IgG physisorption	8437	13040	91318	2.3,^e^ 1.9^d^	2.3^c^	10.9^c^	84.9^c^	0.03	1.02
after IgG chemisorption	87821	9689	64816	28.7^d^	1.7^c^	7.9^c^	61.7^c^	0.39	1.40

a Calculated using the integrals evaluated from zero to the sputter time indicated by the half-maximum of the Si^–^ signal.

b Converted from the mole fraction values (0; 0.028; 0.243; 0.729; respectively, determined based on the XPS data), using the *M*_n_ and ρ values (see Table S3 for the mers, *M*_n_ = 208.1 g/mol and ρ = 1.64 g/cm^3^ for the ATRP initiator bound to APTES, *M*_n_ = 110 g/mol and ρ = 1.37 g/cm^3^ for amino acids).

c Rescaled, using the averaged intensity of C_4_H^–^ and C_4_H_5_O_2_^–^ ions, with respect to that of the as-prepared coating.

d Obtained by subtracting from unity the values for the other 3 components.

e Rescaled, using the relative net intensity of CNO^–^ ions obtained by subtracting the value of the as-prepared coating.

f Induced by the reduction of the total average volume fraction of brush components (OEGMA, MAA, APTES-ATRP).

**Figure 6:**
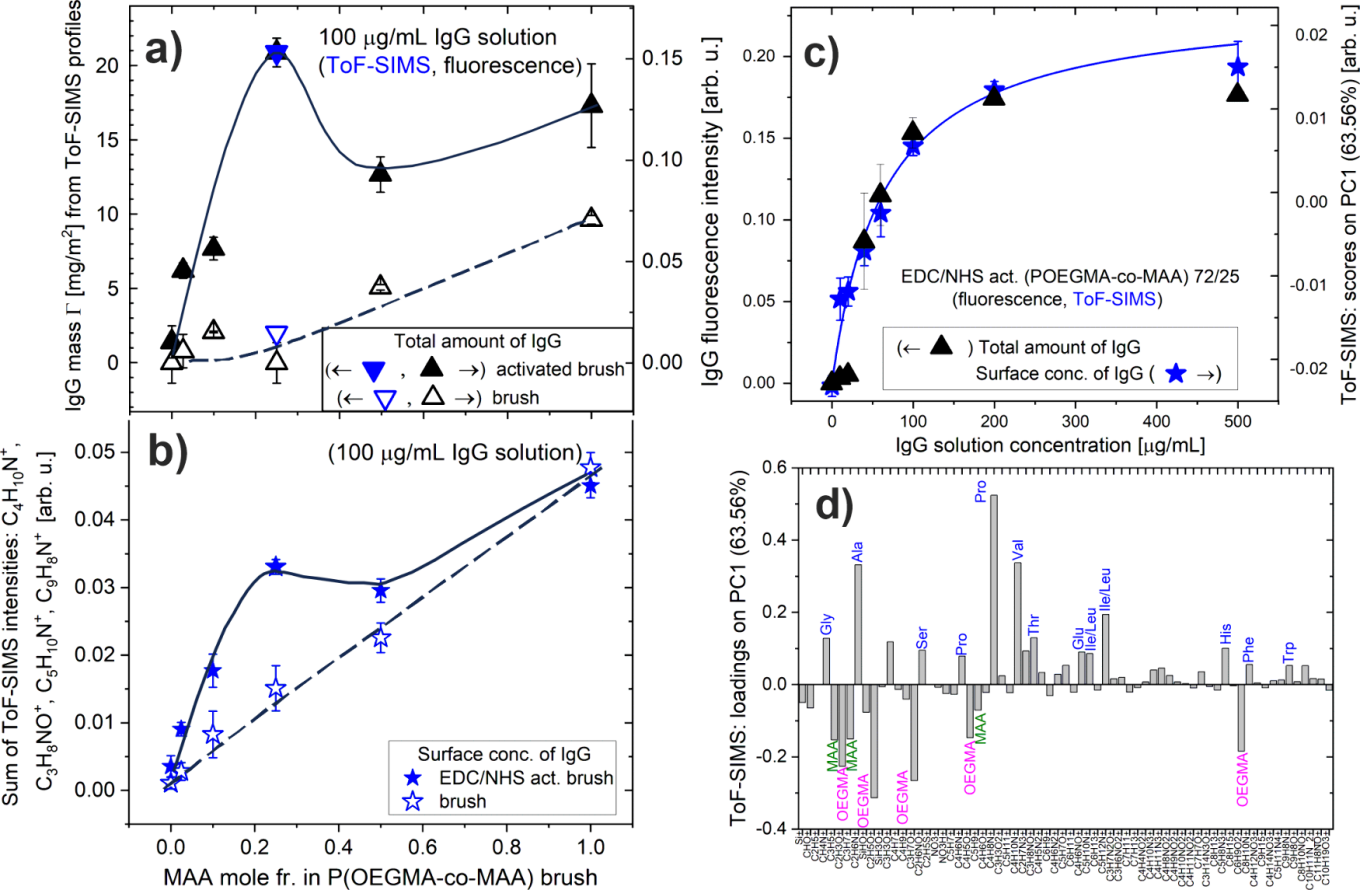
Total amounts (triangles) and surface concentrations (stars) of IgG antibody within brush coatings: (a, b) P­(OEGMA_1–*x*_-*co*-MAA_*x*_) (analyzed as a function of the mole fraction *x* of MAA) and (c, d) P­(OEGMA-*co*-MAA) 75/25 (examined as a function of the concentration of the applied IgG solution), after physical adsorption (open symbols) or covalent immobilization (solid symbols), the latter enabled by an earlier brush activation using the EDC/NHS coupling procedure. Data obtained from ToF-SIMS and fluorescence microscopy (blue and black symbols, respectively) are collated. (a, b) IgG fluorescence intensity and ToF-SIMS data compared for physical adsorption and covalent immobilization. Arbitrary fluorescence units of (a) and (c) are scaled to match the absolute value Γ of the IgG mass per area (a, left axis), obtained from ToF-SIMS profiles (Figure 4) for the P­(OEGMA-*co*-MAA) 75/25 brush and 100 μg/mL IgG solution. (c, d) Results of the multivariate PCA analysis of ToF-SIMS data, with PC1 differentiating between the characteristic composition of the protein and the activated polymer brush, used to plot surface coverage with protein, described with the Langmuir model (solid line) and compared with the fluorescence IgG intensity.

#### 3.2.2 Protein Conjugation and Protein Resistance of Copolymer Brushes

To optimize P­(OEGMA-*co*-MAA) copolymer brush coatings in terms of biosensing applications, we strived to maximize the covalent immobilization capacity of the functional protein for detection efficiency, while simultaneously reducing nonspecific protein adsorption for a lower background. The immobilization of the IgG antibody, acting as a detecting molecule in immunosensors, was examined for P­(OEGMA-*co*-MAA) brush coatings with *different compositions* and at *various protein solution concentrations*. The load of immobilized IgG was evaluated by the *total protein amount* provided by florescence microscopy and ToF-SIMS depth profiling, and indicated by the surface protein concentration determined with ToF-SIMS working under static mode conditions. To covalently attach the antibody within the brush, we applied EDC/NHS coupling chemistry. To evaluate antifouling properties, we determined the level of physical adsorption for nonactivated coatings.

First, the successful activation of the carboxyl groups of the MAA segments with EDC/NHS, forming NHS ester species (Figure 5a,b), was evidenced by ToF-SIMS analysis. High mass resolution ToF-SIMS spectra revealed a 4-fold increase in intensity of the C_4_H_14_NO_3_^+^ (*m*/*z* = 124) ion signal derived from the *N*-hydroxysuccinimide group after EDC/NHS activation of PMAA and P­(OEGMA-*co*-MAA) 75/25 brushes (Figure S5). Second, after covalent binding or physical adsorption of the IgG antibody (described in Section 2.2, Figure 5c,d), fluorescence micrographs were recorded for EDC/NHS activated and nonactivated P­(OEGMA-*co*-MAA) brush coatings with different composition. The same concentration of IgG solution (100 μg/mL, 150 mM, pH 7.4) was applied to all brush coatings and images were taken under identical conditions (magnification and exposure time). To compare the *total amount of immobilized protein*, semiquantitative micrograph analysis was performed using Minkowski measures, as previously described.^37^ Fluorescence intensities determined in this way (black solid triangles in Figure 6a and Figure S6) show that the amount of IgG antibody *bioconjugated* to the EDC/NHS activated brushes increases with MAA content, induced by an increasing number of protein coupling sites (Figure 5d). As expected, for the POEGMA brush, the amount of protein is negligible, as there are no activated covalent binding groups and the POEGMA polymer is resistant to protein.^31,54^ This property results from the combined impact of steric hindrance and hydration shell related to oligo­(ethylene glycol) side chains of the OEGMA segments^55^ (Figure 5c,d). Surprisingly, the fluorescence intensity of the IgG antibody attached covalently within the brush reaches a maximum for the MAA mole fraction *x* = 0.25, and is even higher for this copolymer composition than for the PMAA homopolymer. This observation may be partly related to the difference in brush thickness, with *h*_dry_ ∼ 25 nm for PMAA and *h*_dry_ ∼ 55 nm for P­(OEGMA_1–*x*_-*co*-MAA_*x*_) copolymers with *x* not greater than 0.25 (Table 1). To shed more light on this issue, the *protein concentration on the surface* of the EDC/NHS activated P­(OEGMA-*co*-MAA) brush coatings was compared with ToF-SIMS (solid stars in Figure 6b). The sum of ToF-SIMS signals characteristic for IgG^56^ (amino acid ion fragments C_4_H_10_N^+^ (Val), C_3_H_8_NO^+^ (Ser), C_5_H_10_N^+^ (Lys) and C_9_H_8_N^+^ (Trp)^57^) was used, which have the lowest contributions from bare brush coatings. Similarly to fluorescence microscopy (cf. Figures 6a and 6b), ToF-SIMS data reveal an increase in protein surface concentration with the MAA molar fraction and an undetectable amount of protein on the POEGMA coating. However, instead of a clear maximum, the P­(OEGMA-*co*-MAA) 75/25 brush coating is distinguished by the beginning of a plateau in protein surface composition. This appears to reflect saturation in the total protein mass loaded per brush volume, with Γ/*h* of 0.39 g/cm^3^ estimated with ToF-SIMS profiling and corresponding to several IgG monolayers. Indeed, the corresponding total protein mass per surface area Γ of 21.5 mg/m^2^ (blue solid triangle in Figure 6a), is much higher than that of the IgG monolayer on APTES-activated silicon (Γ ∼ 2.5 mg/m^2^ ^58^). A further increase of Γ/*h* with the molar fraction of MAA in the copolymer brush (Figure 6b) is more limited and is related to the optimized spatial use of IgG molecules.

To evaluate the *physical adsorption* of the IgG antibody by the polymer brush coatings with different composition, we again complement fluorescence microscopy with ToF-SIMS (in static mode). While for the fluorescence method, the sensitivity to a low amount of adsorbed protein can be limited due to the need to maintain a constant exposure time for all samples, ToF-SIMS has a detection limit for adsorbed protein in the ng/cm^2^ range.^59,60^ Therefore, the results of ToF-SIMS analysis (open stars in Figure 6b) are more sensitive to low protein coverage than fluorescence microscopy (black open triangles in Figure 6a). Both data sets show that the load of physically adsorbed IgG antibody in nonactivated brush coatings starts from zero for the POEGMA brush (resistant to proteins^31,54^ ), and increases with the MAA molar fraction *x*, to reach at *x* = 0.25 total protein mass per surface area Γ = 1.4 mg/m^2^, as determined with the ToF-SIMS profiles (open blue triangle in Figure 6a), and at *x* = 1.0 (PMAA) becomes comparable to that of chemisorbed IgG (Figure 6b) with total mass loaded per brush volume Γ/*h* exceeding 0.4 g/cm^3^. Higher protein loads by PMAA brushes (converted from PtBMA) have recently been reported and are related to the hydrogen bonding with the protonated carboxylic acid groups of MAA, for solution pH < p*K*_a_ of the brush, or with electrostatic bond interactions for deprotonated MAA mers and positively charged proteins, for the protein isoelectric point > solution pH > p*K*_a_ of the brush.^61^ In our case, the pH 7.4 buffer applied induces interactions between negatively charged MAA brush segments^41^ and electric dipole moments formed at antibodies,^62^ which are responsible for our results (Figure 5c). These electrostatic forces, tuned by the fraction of MAA mers in the copolymer brush, lead to an increase in the load of IgG protein that is more gradual than that caused by bioconjugation (Figure 6b).

The load of protein immobilized in the P­(OEGMA-*co*-MAA) brush can be controlled by not only the composition of the brush but also *the concentration of protein solution*. The adsorption isotherm was determined for the EDC/NHS activated P­(OEGMA-*co*-MAA) 75/25 brush coating using IgG solutions with concentrations ranging from 10 to 500 μg/mL. The *total amount* of immobilized IgG, examined with fluorescence microscopy using the Minkowski measure as described above, is compared to the *surface concentration* of IgG, determined with ToF-SIMS (Figure 6c,d). This comparison is possible as a result of a constant with depth brush composition. PCA analysis was applied to the ToF-SIMS data, formed by 68 ion fragments characteristic of the OEGMA and MAA segments, and amino acids, normalized to the total intensity of the ions, and are listed in Figure 6d. The first principal component, PC1, which captures 63.56% of the total variance in the data set, distinguishes the samples based on protein coverage, as indicated by the loadings plot (Figure 6d). The secondary ions derived from copolymers load PC1 in the negative direction, while those characteristic for amino acids load in the positive direction. Therefore, the mean values of the PC1 scores can be used as a measure of the IgG surface density obtained from solutions of varying concentration. The adsorption isotherms, which present the fluorescence intensity and the mean value of the PC1 scores, are shown in Figure 6c. Here, for the constant composition and thickness of the copolymer brush, the total amount of IgG agrees well with the surface IgG concentration. The amount of immobilized IgG increases rapidly with the concentration of the solution and begins to saturate at a concentration greater than 200 μg/mL. The affinity constant, achieved by fitting the Langmuir model to the ToF-SIMS data, is about ∼2.3 × 10^6^ 1/M. These results indicate that the total protein mass loaded per brush volume Γ/*h* can be adjusted between zero and 0.47 g/m^3^ for IgG antibodies and P­(OEGMA-*co*-MAA) 75/25 brush coatings.

### 3.3 Immunorecognition and State of Antibodies Bioconjugated to P­(OEGMA-*co*-MAA) Brush Coatings

#### 3.3.1 Molecular Recognition and Nonspecific Binding of Antibody-Conjugated Brushes

Biosensing applications of polymer brush coatings based on the specific interaction between an antigen and an immobilized antibody require a high binding affinity. The reduction in immobilized antibody activity may result from denaturation of its three-dimensional structure, a steric hindrance, or an unfavorable orientation that affects the access to the binding sites. For the polymer brushes with bioconjugated antibodies studied here, the amino acid amide bonds with the EDC/NHS activated MAA segments (Figure 7a) can alter the functions mediated by the Fab and Fc fragments, in addition to the effects due to steric hindrance, altered conformation, and orientation of IgG. To examine the antigen binding capacity of antibodies (goat anti-rabbit IgG) covalently immobilized on P­(OEGMA-*co*-MAA) brush coatings, an immunoreaction with rabbit IgG (rIgG) antigen was performed. To prevent nonspecific antigen binding, before immunoassay, IgG antibody-functionalized coatings were immersed in a hydroxylamine solution to hydrolyze unreacted NHS ester groups. The preservation of antifouling properties of copolymer brush coatings after subsequent steps of EDC/NHS activation, immobilization of IgG, and hydrolyzation of unreacted NHS groups was examined by incubation with the solution of fluorescence-labeled BSA protein (2 mg/mL in PBS). Fluorescence microscopy was applied to compare an amount of immobilized antibody, adsorbed BSA, and bound antigen in polymer brush coatings with different composition. Fluorescence micrographs of the IgG antibody, the BSA protein, and the rabbit IgG antigen were recorded for separate samples but with the same exposure time as described in Section 2. As shown in Figure 7b, the load of antibody in the copolymer brush (filled columns) has the maximum for the MAA mole fraction of 0.25 in addition to a monotonic increase with MAA content, reproducing the results shown in Figure 6a. The adsorption of BSA (gridded columns) was detected only on PMAA homopolymer coatings, which confirms the antifouling properties of the P­(OEGMA-*co*-MAA) coatings after functionalization with the IgG antibody. Similarly to that for the IgG antibody, an amount of bound rIgG antigen (striped columns) shows a clear maximum for the 0.25 MAA mole fraction in the P­(OEGMA-*co*-MAA) copolymer brush, in addition to no major differences in the rIgG fluorescence intensity for other brush compositions. For copolymer brushes with a high OEGMA content (MAA molar fraction 0.025 and 0.1), the fluorescence intensity of the rIgG antigen is higher than that recorded of the IgG antibody, indicating the high activity of the immobilized antibodies. Then, a decrease in antigen binding ratio is observed with increasing MAA content. One of the reasons for this decrease in antigen binding efficacy may be steric hindrances that increase with the density of antibody mass in brush volume. However, the antigen to antibody binding ratio is higher for the P­(OEGMA-*co*-MAA) 75/25 copolymer than for the PMAA homopolymer, suggesting the involvement of other factors related to changes in *interfacial protein state*, i.e., IgG orientation, conformation, or the amount of NHS-bound/unbound amino acids.

**Figure 7:**
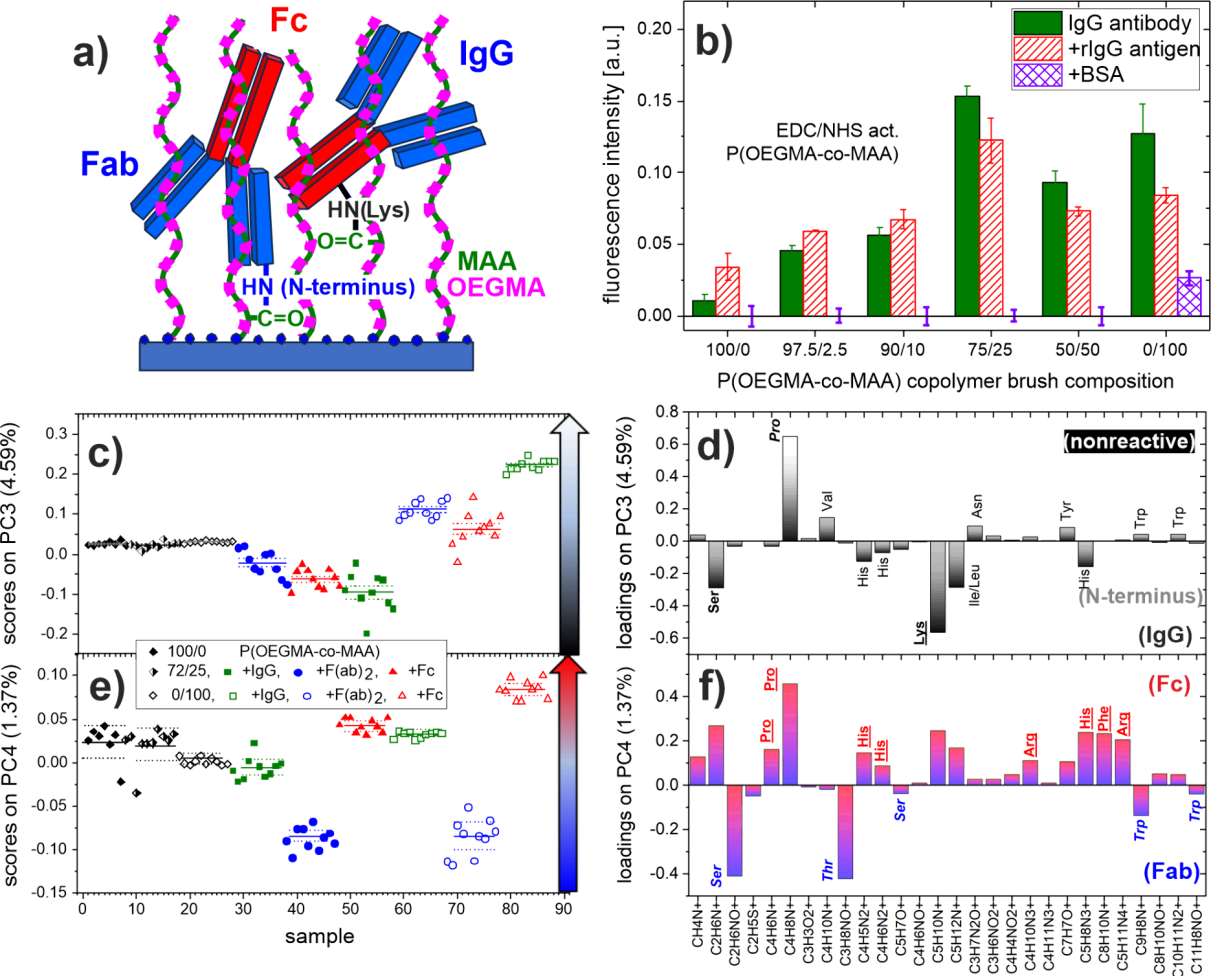
(a) Schematic presentation of IgG antibodies covalently attached to the EDC/NHS activated MAA segments of P­(OEGMA-*co*-MAA) brushes, (b) nonspecific adsorption and antigen binding (fluorescently labeled BSA and rIgG, respectively) to such bioconjugated brushes as a function of brush composition, and (c–f) the *interfacial protein state* of IgG antibody at the brush surface determined with the PCA analysis of ToF-SIMS data. (c, e) PC3 and PC4 scores, plotted for individual measurements, with the differences (marked with vertical arrows) between the IgG antibodies attached to PMAA and P­(OEGMA-*co*-MAA) 75/25 brushes in (c) *residue involvement in linkage formation* (within the brush) with MAA segments and (e) *dominant antibody orientation*. Data for the reference layers of the antibody fragments (F­(ab)_2_ and Fc) and the bare POEGMA coating are included. (d) Loadings plot for PC3: Negative PC3 loadings are from residues (exposed to the surface) ready to form bonds with NHS esters, dominated by lysine (distributed throughout the IgG molecule), with smaller contributions from other amino acids (only when located at the N-terminus). Positive loadings are mainly from proline, which is nonreactive to NHS because of its secondary amine. (f) Loadings plot for PC4: ion fragments of amino acids more abundant in the Fab domain (blue italic) load in the negative direction, while positive loadings are from amino acids with higher content in the Fc domain (red underlined).

#### 3.3.2 Interfacial Protein State of Brush-Conjugated Antibodies

An Y-shaped IgG molecule, consisting of the Fc trunk and two Fab domains with antigen binding sites, can adopt different *orientations at the surface* (i.e., flat-on, side-on, tail-on, and head-on) that vary in access to binding sites. Consequently, the orientation of the immobilized IgG antibodies determines the efficiency of the assay. ToF-SIMS is a powerful *surface-sensitive* and *chemically specific* technique suitable for the direct analysis of dominant antibody orientation, based on examination of the outermost region of adsorbed IgG antibodies and resolution of amino acid composition between the Fab and Fc domains.^8,63^ ToF-SIMS with PCA has been successfully applied to compare the dominant orientation of IgG molecules adsorbed on different types of surfaces, such as SAM-modified gold and silicon supports,^58,62,64–66^ polymer^30,66–68^ and protein layers.^69,70^ Moreover, an analysis of antibody orientation as a function of its surface density Γ can provide the proportion of densely adsorbed molecules adopting coexisting head-on and tail-on orientations.^58,62,71^ Despite advances in antibody orientation analysis, it remains challenging to perform the analysis for organic substrates with complex structure, such as copolymer brushes. Methods based on the determination of antigen binding efficiency are indirect and inaccurate, while the application of ToF-SIMS requires a precise selection of reference samples and careful data interpretation due to overlapping signals from protein and other organic (macro)­molecules.

In this work, ToF-SIMS supported with PCA was applied to examine the *interfacial protein state*, including dominant orientation, of the IgG molecules immobilized at the surface of polymer brushes, compared for copolymer P­(OEGMA-*co*-MAA) and PMAA homopolymer coatings. P­(OEGMA-*co*-MAA) 75/25 was selected based on its optimal balance between efficient antibody loading and protein resistance properties. The PCA model was developed based on ToF-SIMS data recorded from P­(OEGMA-*co*-MAA) 75/25 and PMAA coatings with immobilized IgG antibody, and F­(ab)_2_ and Fc antibody fragments, as well as from POEGMA, PMAA and P­(OEGMA-*co*-MAA) 75/25 bare coatings. To detect subtle differences in the IgG protein state, only the characteristic amino acid ion fragments (listed in Figure 7d,f) were included in the PCA analysis and the intensity was normalized to the sum of these peaks. The results of the PCA analysis are presented in Figure 7c–f and Figure S7. Due to the contributions of ion signals from the brush to the signals characteristic for amino acids, in the developed PCA model, the first (PC1) and the second principal component (PC2) that capture the majority of variance (63.01% and 26.06%, respectively) reflect the combined information on brush surface coverage with proteins and polymer brush composition (see Figure S7a–c). In the PC1 vs PC2 score plot (Figure S7a), the IgG (and its fragments), PMAA and POEGMA data points are centered around three points, which can be envisioned as the vertices of a triangle (see ref 56 for a similar analysis), with the P­(OEGMA-*co*-MAA) data located on the longest side of the triangle. Due to the orthogonality of the Principal Components, the *interfacial protein state* of our interest for IgG is described by PC3 and PC4, which capture, respectively, 4.59% and 1.37% of the variance. PC3 clearly separates proteins (IgG, Fc, and F­(ab)_2_ domains) attached to PMAA and P­(OEGMA-*co*-MAA) 75/25 brushes (Figure 7c). This separation was initially attributed to differences in *protein denaturation*, as hydrophilic polymer chains are known to reduce conformational changes.^3,72^ However, PC3 loadings do not correlate with amino acid hydrophilicity, disproving that hypothesis^6,30,73^ (see Figure S7d). In turn, PC3 loadings correlate with the *participation of amino acids in the formation of amide bonds* with the EDC/NHS activated MAA segments (see Figure 7d). Negative PC3 loadings are dominated by lysine (with ε-amine), ready to form such bonds for residues distributed throughout the IgG molecule, with smaller contributions from other amino acids (with α-amine; such as Ser, Ile/Leu, His) that can form bonds with NHS esters only when located at the N-terminus.^74,75^ Positive PC3 loadings are essentially from proline, which is not reactive to NHS due to its secondary amine. The corresponding values of PC3 scores (Figure 7c) are positive for the IgG antibody (and its fragments) on the PMAA brush, indicating reduced *surface exposure* of NHS reactive amino acids (mainly Lys) and increased exposure of nonreactive Pro on the sample surface. This may be related to an enhanced bond formation with MAA mers (*within the brush*) due to the high density of carboxylic acid groups (223 MAA mers/nm^2^). In contrast, the P­(OEGMA-*co*-MAA) 75/25 brush, with a lower density of coupling sites (56 MAA mers/nm^2^) and the presence of hydrophilic OEGMA chains, allows greater protein mobility and less constrained arrangement. Finally, the PC4 scores plot differentiates in the same way the data points of two pairs of reference samples, with fragments Fc (triangles) and F­(ab)_2_ (circles) immobilized within both PMAA and P­(OEGMA-*co*-MAA) brushes (Figure 7e). The corresponding loading plot on PC4 (Figure 7f) shows that PC4 is positively loaded by secondary ions derived from amino acids abundant in the Fc domain (Pro, His, Arg and Phe), and negatively loaded by those abundant in the Fab domain (Ser, Thr, Trp). This is in agreement with our previous ToF-SIMS studies of the same antibody immobilized on SAM-modified silicon.^58,62,71^ Therefore, the PC4 scores obtained for the IgG antibody attached to the PMAA and P­(OEGMA-*co*-MAA) coatings can be considered as an indicator of the *dominant orientation of IgG molecules* on the surface of these coatings (Figure 7e). The increased scores on PC4 reflect the increased fraction in the footprint area of the entire IgG molecule taken by its exposed Fc domain. The values of the PC4 scores determined for IgG in the PMAA coatings (open squares) are higher than those of P­(OEGMA-*co*-MAA) 75/25 layers (solid suqares), indicating the dominant orientation of IgG with the more exposed Fc domain. This same conclusion can be drawn from a more rigorous approach comparing for each coating separately the PC4 score values for IgG with respect to the difference between the values for the Fc and F­(ab)_2_ fragments (∼78% for PMAA and ∼63% for the P­(OEGMA-*co*-MAA) 75/25 coating). IgG molecules are attached to brush chains with a high mass loading per brush volume (close to 0.4 g/cm^3^), which corresponds to a high *surface* density for an IgG-thick^76^ brush layer. Therefore, coexisting vertical orientations (head-on and tail-on) are expected for IgG antibodies at the brush surface.^58,62^ Based on the analysis of PC4 scores, a higher proportion of molecules adapting an active tail-on orientation is concluded on the surface of the P­(OEGMA-*co*-MAA) 75/25 than the PMAA brush coatings. This feature, causing an increase in antigen binding efficiency as discussed above, is an additional advantage of the developed P­(OEGMA-*co*-MAA) copolymer brush coatings.

The dominant orientations of IgG antibodies at the surface of different brush coatings result from the interplay of IgG interactions with other antibodies and with other (macro)­molecules (Figure 5d). For adsorbed IgG antibodies (from a pH 7.4 buffer, as here) on the SAM surfaces of NHS-silane, amino-silane (APTES), and glutaraldehyde-activated APTES (APTES/GA), no effective surface charge was concluded for the hardy protonated APTES, protonated GA and nonhydrolyzed NHS groups.^58,62^ Therefore, the electric dipoles formed at antibodies between the Fc and F­(ab)_2_ fragments (with isoelectric points below and above, respectively, the pH of the solution) promote the opposite orientation of neighboring antibodies physiosorbed at APTES silane.^58^ In turn, the 3:1 proportion of IgG molecules with head-on to tail-on orientation chemisorbed at APTES/GA silane resulted from random immobilization through Lys residues (with ε-amine) with head-on orientation promoted in addition by conjugation through more reactive (owing to lower p*K*_a_) α-amine of the N-terminus.^62^ Interestingly, NHS-silane-chemisorbed IgG antibodies show an antiparallel arrangement of IgG molecules, similar to APTES, related to slow physisorption that precedes covalent binding and allows dipole–dipole alignment. The latter situation is modified here for antibodies conjugated to polymer brushes, since negatively charged carboxylate groups are expected along polymer chains in light of the acid dissociation constant determined for the PMAA brush (pH > p*K*_a_ 6.5).^41^ Electrostatic interactions of negatively charged MAA segments with antibody dipoles would promote the head-on IgG orientation. This tendency would be weaker for a lower MAA fraction in the brush chains, and therefore a higher proportion of molecules adapting an active tail-on orientation is expected on the surface of the P­(OEGMA-*co*-MAA) 75/25 rather than the PMAA brush coatings, as observed. Disruption of electrostatically promoted head-on IgG alignment may be enhanced by greater protein mobility and less constrained arrangement, which is expected for a lower fraction of covalent-coupling MAA sites in the polymer brush.

### 3.4 Biocompatibility of P­(OEGMA-*co*-MAA) Brush Coatings

The cytocompatibility of P­(OEGMA-*co*-MAA) copolymer brush coatings is a key issue for their biomedical applications. The impact of the polymer brush on cells was verified by culturing dermal fibroblasts on P­(OEGMA-*co*-MAA) 75/25 coatings. To demonstrate their potential as protein functionalized substrates for cell study, we also examine P­(OEGMA-*co*-MAA) coatings with immobilized fibronectin. The growth, morphology, and viability of the cells were traced for 5 days. Representative fluorescence images taken after 72 h of culture on the glass substrate, on the P­(OEGMA-*co*-MAA) 75/25 brush, and the fibronectin functionalized copolymer brush are presented in Figure 8a–c. On all substrates, cells are flattened with spindle-like shapes typical of fibroblasts, indicating favorable culture conditions. After 72 h, the number of cells on the P­(OEGMA-*co*-MAA) brush is comparable to that observed on the reference glass substrate (Figure 8d). For comparison, homopolymer brush coatings showed a reduction in the number of cells for POEGMA (by approximately 65%)^27^ and an increase in the number of cells for PMAA (by ∼30%)^41^ relative to the control substrate. Cell viability on P­(OEGMA-*co*-MAA) 75/25 coatings was determined after 24, 72, and 120 h of culture with the MTT colorimetric test, which provided information on mitochondrial activity (Figure 8e). For both the copolymer brush coating and the silica substrate, cell viability increases monotonically with culture time, with slightly lower values observed for the copolymer coating. In turn, single-component POEGMA and PMAA brushes showed after 72 h reduced viability (by ∼ 30%) and improved viability (by ∼ 18%), respectively, compared to the control.^27,41^ These results indicate that P­(OEGMA-*co*-MAA) brush coatings support cell culture and exhibit biocompatibility properties intermediate between those of PMAA and POEGMA brushes. Furthermore, the functionalization of the copolymer brushes with fibronectin led to a rapid increase (four times) in the number of cells (Figure 8d). This reflects the improved interactions between cells and the substrate due to the presence of the extracellular matrix protein.^77–79^ The pronounced effect on cell growth indicates preservation of conformation and biological activity^79^ by fibronectin immobilized on P­(OEGMA-*co*-MAA) brush coatings.

**Figure 8:**
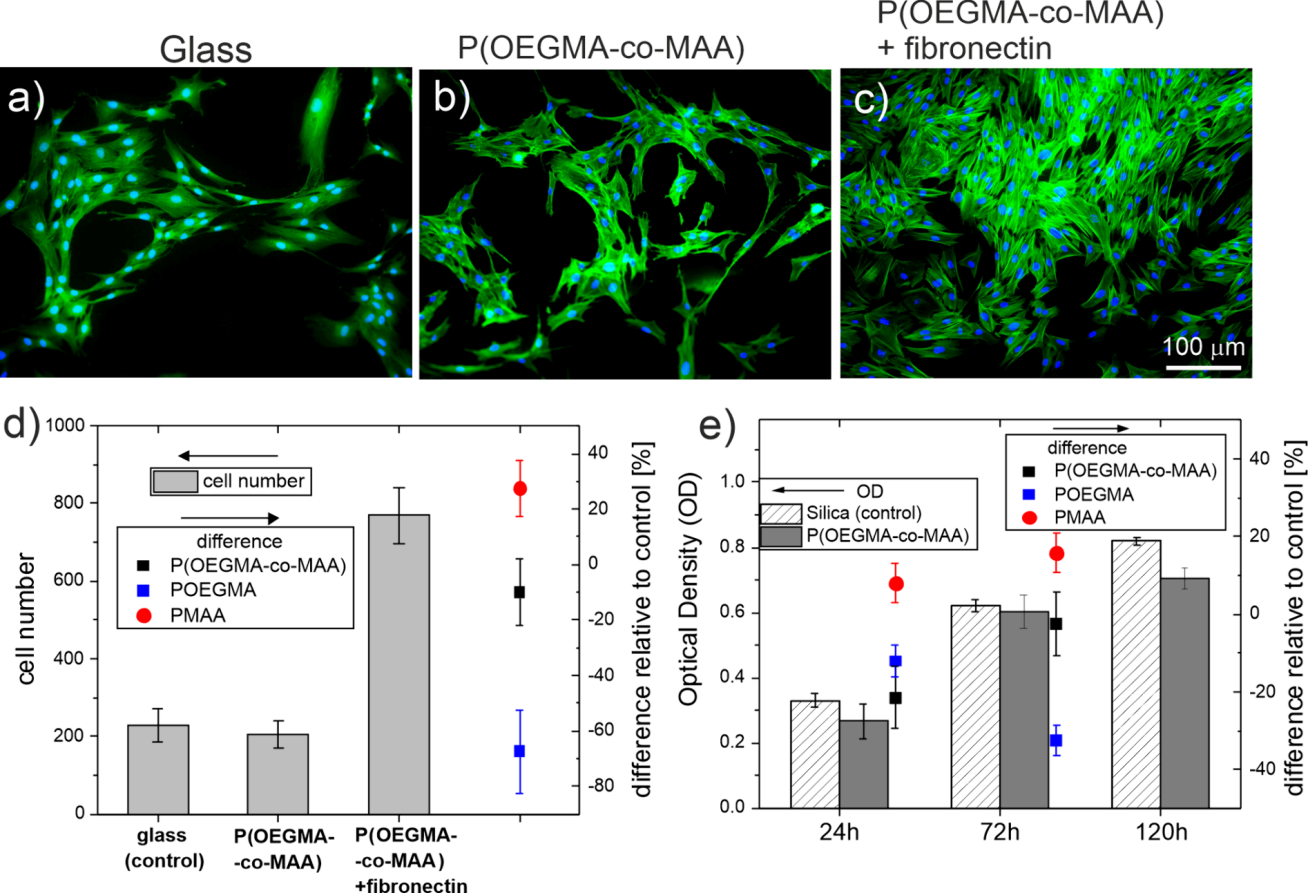
Culture of human dermal fibroblasts on P­(OEGMA-*co*-MAA) 75/25 brush coatings. (a–c) Fluorescence images (cytoskeleton, green; nuclei, blue) of human dermal fibroblasts cultured on glass, copolymer brush coating and copolymer brush coating with immobilized fibronectin protein visualized at 72 h of cell culture. (d) Number of cells on different substrates calculated after 72 h of cell culture. (e) Results of the MTT test performed after 24, 72, and 120 h of cell culture. Data for POEGMA and PMAA homopolymer brush coatings (marked by points) are presented in relation to control substrates according to refs 27 and 41, respectively.

## 4 Conclusions

Novel P­(OEGMA-*co*-MAA) random copolymer brush coatings were synthesized using ATRP polymerization on silicon surfaces modified with APTES. A series of brush coatings prepared varied in the composition of the OEGMA and MAA segments, preventing nonspecific protein adsorption and providing bioconjugation sites for functional proteins, respectively. Multitechnique characterization was carried out to determine the composition, morphology and wettability, thickness and grafting density of the copolymer brushes. XPS chemical bonding analysis, corrected for the presence of the ATRP initiator and adventitious carbon, revealed the molar MAA fraction in the copolymer brush equal to that of the reaction mixture. Furthermore, ToF-SIMS analysis of the molecular surface composition confirmed the XPS findings, while depth profiling demonstrated a uniform composition through the brush, confirming a successful random copolymerization.

The P­(OEGMA-*co*-MAA) brushes were further evaluated for their capacity to immobilize IgG antibody. Fluorescence microscopy and ToF-SIMS were used to determine the total amount and surface concentration of IgG molecules, respectively, for both their covalent attachment to EDC/NHS-activated brushes and their physical adsorption to nonactivated ones. Unlike previous studies on random copolymer brushes that combine antifouling ethylene glycol methacrylate chains and monomers containing side chains for protein coupling,^23,24^ this work systematically investigated different copolymer compositions. A 0.25 molar fraction of MAA (∼168 OEGMA mers/nm^2^ and ∼56 MAA mers/nm^2^) was found to be optimal with the maximal load of the immobilized IgG antibody, while maintaining low fouling properties. ToF-SIMS depth profiling, demonstrated here for the first time for protein-functionalized polymer brushes, clearly evidenced a uniform IgG distribution through the brush thickness. Furthermore, the depth profiles of the characteristic protein, MAA and OEGMA ions allowed the estimation of the total IgG mass per brush volume, reaching ∼0.4 g/cm^3^ for the P­(OEGMA-*co*-MAA) 75/25 brush activated with EDC/NHS. The corresponding mass per surface area was nine times higher than that of the IgG monolayer. In contrast, only ∼0.03 g/cm^3^ was estimated for the physically adsorbed IgG protein within the nonactivated brush. The protein load in the brushes can be controlled by their composition and the concentration of the applied protein solution.

Indeed, the corresponding total protein mass per surface area Γ of 21.5 mg/m^2^ (blue solid triangle in Figure 6a), is much higher than that of the IgG monolayer on APTES-activated silicon (Γ ∼ 2.5 mg/m^2^ ^58^).

The 0.25 MAA molar fraction in P­(OEGMA-*co*-MAA) also provided the highest amount of bound antigen for the IgG antibody functionalized brush, with an antigen binding ratio higher than that of the single component PMAA coating. This observation was further investigated by PCA analysis of the ToF-SIMS data, and it was related to the different *interfacial antibody states* for the P­(OEGMA-*co*-MAA) and PMAA brushes. The principal component PC3 differentiated the IgG proteins attached to both brushes in terms of surface-exposed *reactive residues* (mainly lysine) *and residues nonreactive* to the EDC/NHS activated MAA segments (proline). The surface composition of the protein-functionalized PMAA brush was enhanced in proline compared to that of the copolymer brush, reflecting a higher abundance of protein residues covalently linked with the MAA segments, resulting from their higher areal density. In addition, PC3 loadings disproved the hypothesis of *conformation* differences between proteins in both brushes. In turn, the principal component PC4, which reflects the dominant antibody *orientation*, revealed the higher proportion of molecules in an active tail-on arrangement on the P­(OEGMA-*co*-MAA) brushes. Promotion of the inactive head-on orientation on the PMAA brush through electrostatic interactions is again related to the higher areal density of negatively charged MAA segments. The introduction of hydrophilic OEGMA chains and the reduced areal density of the protein coupling species in the P­(OEGMA-*co*-MAA) copolymer brush reduce the constraint on the molecular arrangement, leading to enhanced biological activity.

Finally, the biocompatibility of the copolymer brush coatings was confirmed by human fibroblast cell culture, broadening the potential applications of the developed coatings. In particular, the biocompatibility of the developed coatings can be further promoted by their bioconjugation with functional proteins, as demonstrated for fibronectin.

## Supplementary Material



## Notes

Additional information and data on (i) scheme of the functionalization of silicon substrates with P­(OEGMA-*co*-MAA) brush coatings; (ii) analysis of the copper contamination in the copolymer brush coatings; (iii) details on determination of the XPS atomic concentrations; (iv) details on determination of the grafting density of polymer chains based on the thickness of the brush in dry and swollen state; (v) examination of the copolymer brush coatings uniformity; (vi) morphology and wettability of copolymer brush coatings; (vii) ToF-SIMS examination of the EDC/NHS activation of the copolymer brush; (viii) total amounts of the IgG antibody in the P­(OEGMA_1–*x*_-*co*-MAA_*x*_) brush coatings analyzed as a function of the surface density of both mers; (ix) ToF-SIMS and PCA analysis of the state of the IgG antibody immobilized within copolymer brushes (PDF)
