# Supplementary material for: Integrating Protein Resistance and Bioconjugation in P(OEGMA-co-MAA) Brushes for Biosensing and Cell Culture: ToF-SIMS Profiling and Antibody Characterization
Source: ACS Appl Mater Interfaces. 2025 Dec 22;18(1):3122–39. doi: 10.1021/acsami.5c19592 (PMC12781104; doi:10.1021/acsami.5c19592)
Supplement: Supplementary file 1 [file am5c19592_si_001.pdf]

Supporting Information

Integrating protein resistance and bioconjugation in P(OEGMA-co-MAA) brushes for biosensing and cell culture: ToF-SIMS profiling and antibody characterization

Katarzyna Gajos[a], Ostap Lishchynskyi[b], Paweł Dąbczyński[a], Svitlana Tymetska[a,c], Łukasz Bodek[a], Yana Shymborska[a], Natalia Janiszewska[a], Yurij Stetsyshyn[d], Andrzej Budkowski[a]*

[a]M. Smoluchowski Institute of Physics, Jagiellonian University, Łojasiewicza 11, 30-348 Kraków, Poland

[b]Department of Biotechnology, Faculty of Bioscience Engineering, Ghent University, Proeftuinstraat 86, Ghent 9000, Belgium

[c]Jagiellonian University, Doctoral School of Exact and Natural Sciences, Łojasiewicza 11, 30-348 Kraków, Poland

[d]Lviv Polytechnic National University, St. George's Square 2, Lviv, Ukraine

* Corresponding author

E-mail address: katarzyna.gajos@uj.edu.pl

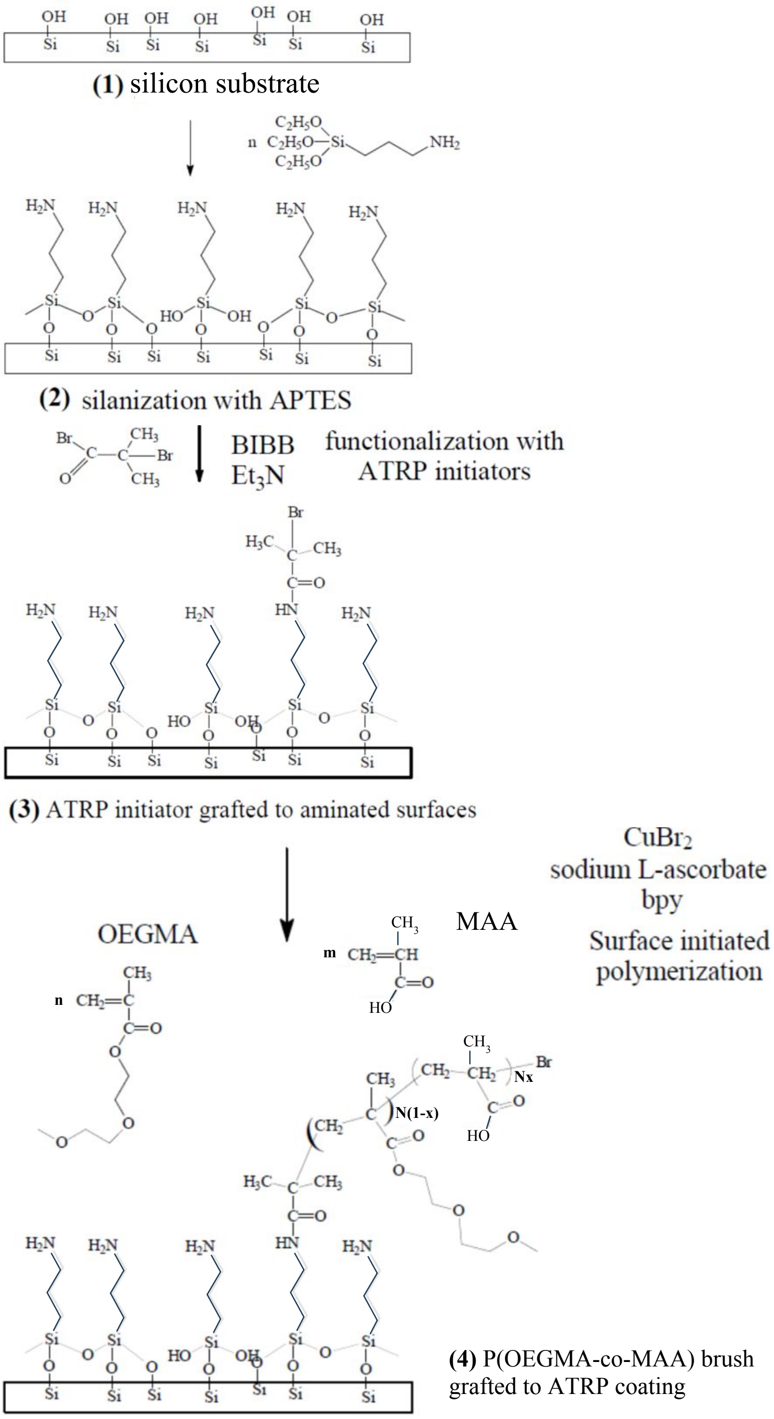

Scheme S1. Functionalization of the silicon surface (1) with amino-terminated APTES film (2), subsequent grafting of the ATRP initiator (3) and polymerization of OEGMA188 and MAA, initiated by the ATRP initiator, resulting in P($OEGMA_{1-x}$-co-MAA_x) brush coatings with varied mole fraction x of MAA (4).

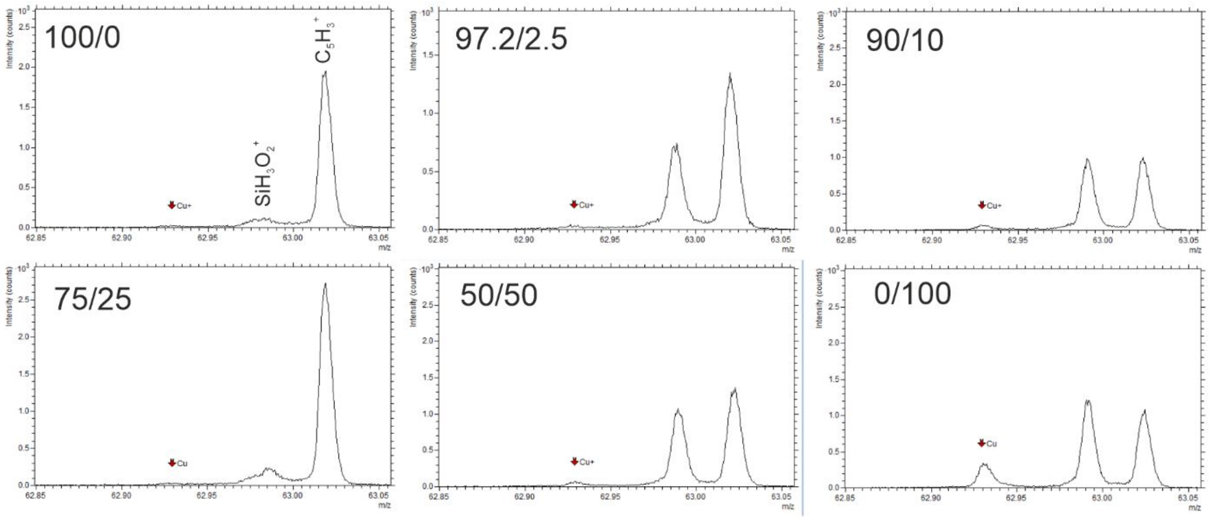

Figure S1. ToF-SIMS analysis of the copper contamination in the synthetized brush coatings.

Table S1. XPS atomic concentrations of nitrogen and carbon with different chemical environments of APTES film with the grafted ATRP initiator, before and after the fabrication of P(OEGMA-co-MAA) n/m brush coatings. For the ATRP initiator samples, carbon concentrations related to the *intrinsic* composition, deduced from the chemical structure (with the mole fraction *y* of APTES molecules functionalized with the initiator), are subtracted from the *measured* values to reveal the composition of *adventitious carbon* (aC) contamination. To correct the carbon concentrations measured for polymer brush samples, we assumed that the contributions due to aC are the same as those for the ATRP initiator samples, but the contributions due to the ATRP initiator are proportional to the N concentration normalized by its value for the samples of the ATRP initiator (see the formulas in the POEGMA 100/0 line).

Sample		XPS composition (at %)					
		N/N(ATRP)	N	C–C [a]	C–N,O [a]	(N–)C=O [a]	O–C=O [a]
ATRP initiator	measured [b]	1.0	7.8(4)	38.4(9)	15.2(2.5)	6.2(6)	0.6(4)
	intrinsic ATRP			*~(2+3y)* 36.6 = *c1*	*~1* 9.0 = *c2*	*~y* [b] 6.2 = *c3*	*~0* 0
	adventitious carbon (aC)	-	-	1.8 = *a1*	6.2 = *a2*	0	0.6 = *a4*
POEGMA 100/0	measured	0.040 = *ni*	0.4(1)	24.1(1.2)	39.9(1)	0.7(1)	7.3(1)
	ATRP+aC			3.3 = *ni*c1+a1*	6.6 = *ni*c2+a2*	0.2 = *ni*c3*	0.6 = *a4*
	intrinsic	-	-	20.8	33.3	0.5	6.7
	measured	0.019	0.15(2)	21.9(1)	39.8(1)	0.18(2)	7.2(2)

P(OEGMA-*co*-MAA) 97.5/2.5	ATRP+aC			2.5	6.4	0.12	0.6
	intrinsic	-	-	19.4<br>*~3*	33.4<br>*~(5-5x)*	0.06<br>*~0*	6.6<br>*~1*
P(OEGMA-*co*-MAA) 90/10	measured	0.022	0.17(3)	23.4(1)	38.2(3)	0.4(3)	7.5(1)
	ATRP+aC			2.6	6.45	0.1	0.6
	intrinsic	-	-	20.8	31.75	0.3	6.9
P(OEGMA-*co*-MAA) 75/25	measured	0.030	0.2(1)	25.7(1)	35.4(4)	0.0(2)	8.5(3)
	ATRP+aC			2.9	6.5	0.2	0.6
	intrinsic	-	-	22.8	28.9	-0.2	7.9
P(OEGMA-*co*-MAA) 50/50	measured	0.195	1.5(2)	31.0(1)	27.1(3)	1.1(2)	8.4(2)
	ATRP+aC			8.9	8.0	1.2	0.6
	intrinsic	-	-	22.1	19.1	-0.1	7.8
PMAA 0/100	measured	0.516	4.0(3)	41.6(2)	12.0(6)	2.8(3)	7.9(1)
	ATRP+aC			20.7	10.9	3.2	0.6
	intrinsic	-	-	20.9	1.1	-0.4	7.3

[a] Binding energies: 284.8 eV (C–C), 286.3 eV (C–N,O), 287.9 eV (N–)C=O) and 288.6 eV (O–C=O).
[b] The ratio of Br and N signals, 0.69(9), reveals the mole fraction *y* of APTES molecules functionalized with the initiator.

Table S2. Composition of OEGMA/MAA reaction mixtures and XPS mole fraction of MAA in synthetized brush coatings P(OEGMA-co-MAA), compared to theoretical predictions for the reactivity ratios $r_1 = 1.03$ and $r_2 = 1.02$ [43].

P(OEGMA-co-MAA) n/m	XPS mole fr. of MAA	Theoretical predictions (mole fr. of MAA)
100/0	0	0
97.5/2.5	0.025	0.025
90/10	0.1	0.099
75/25	0.25	0.249
50/50	0.5	0.501
0/100	1	1

The composition of P(OEGMA-co-MAA) copolymer brush coatings was calculated using Equations (S1) and (S2) [27]:

$$F_1 = \frac{r_1 \times f_1^2 + f_1 \times f_2}{r_1 \times f_1^2 + 2 \times f_1 \times f_2 + r_2 \times f_2^2} \quad \text{(S1)}$$

$$F_2 = 1 - F_1 \quad \text{(S2)}$$

where F_1 and F_2 represent the segment mole fractions of OEGMA and MAA in the copolymer brush coatings, respectively; r_1 and r_2 are the reactivity ratios of OEGMA and MAA; f_1 and f_2 denote the concentrations of OEGMA and MAA monomers in the reaction mixture. Although Equations (S1) and (S2) are used primarily to describe the instantaneous composition of copolymers at low conversion rates (<15%), they can also provide a reasonable estimate of the overall trends in the structure of the grafted brushes.

Table S3. Determination of the coefficients (C_w, C_d) that relate the brush thickness [nm] in the swollen state ($h_{wet} = C_w$ N $\sigma^{1/3}$) and in the dry state ($h_{dry} = C_d$ N σ) with the grafting density σ [chains/cm^2] (based on refs. [41,46]). The coefficient C that relates the density of grafting and the swelling ratio $h_{wet}/h_{dry} = C/\sigma^{2/3}$. Parameters used to determine the (areal number) density of chain segments, equal to ($h_{dry}\rho N_A/M_n$), where N_A is the Avogadro number.

P(OEGMA-co-MAA) n/m	x, MAA mole fr.	M_n, g/mol	ρ, g/cm³	$R_g/N^{3/5}$, nm	C_w[a], $nm^{5/3}$	C_d[b], nm³	C (C_w/C_d), $nm^{2/3}$
100/0	0	188.0	1.08[c]	0.197[d]	0.195	0.289	0.68
97.5/2.5	0.025	185.5	1.08	*0.196*[e]	*0.193*	0.285	*0.68*
90/10	0.1	177.8	1.09	*0.192*[e]	*0.187*	0.271	*0.69*
75/25	0.25	162.5	1.10	*0.184*[e]	*0.174*	0.245	*0.71*
50/50	0.5	137.1	1.14	*0.170*[e]	*0.152*	0.200	*0.76*
0/100	1	86.1	1.29[f]	0.139[g]	0.109	0.111	0.98

[a]Using the relation $C_w = 2\pi^{1/3}(R_g/N^{3/5})^{5/3}$.
[b]Using the relation $C_d = (M_n/\rho)/N_A$, where N_A is the Avogadro number.
[c]Sigma-Aldrich Product Page.
[d]Dalgakiran, E; Tatlipinar, H. *J. Polymer Science B* **2018**, 56, 429–441, doi:10.1002/polb.24555.
[e]For copolymers, the average values based on the composition [$x(a)_{PMAA} + (1-x)(a)_{POEGMA}$] were taken for $a=(R_g/N^{3/5})^{5/3}$.
[f]Polymer Data Handbook: Second Edition, James E Mark (ed.), Oxford University Press, Oxford 1999.
[g]Ref.[41].

Experimental Section S1. Coatings characterization

Water Contact Angle Measurements. For static contact angle measurements, the sessile drop technique was applied using a Kruss EasyDrop instrument (DSA15). Measurements were carried out at room temperature for 'as prepared' coatings. Drops of water (3µL) were placed on the fabricated coatings and imaged immediately with an LCD camera. Contact angles were obtained with circle fitting method and expressed as the average of ten measurements at different spots.

Atomic Force Microscopy. AFM images of copolymer brush coatings were recorded in tapping mode with an Alpha 300R (WITec, Ulm, Germany) microscope. AFM probes with spring constant about 2.8 N/m, tip radius about 7 nm, and resonant frequencies about 75 kHz were

used. All AFM images were analyzed using WSxM software provided by Nanotec Electronica S.L.

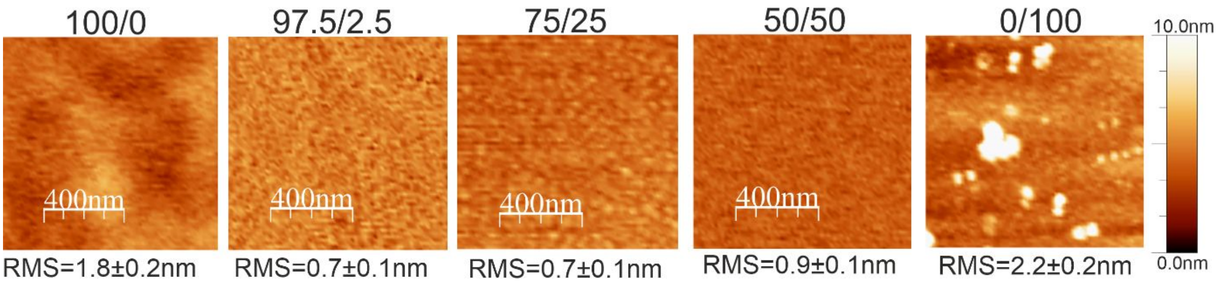

Figure S2. AFM images of the P(OEGMA-co-MAA) brush coatings with different composition. 1μm x1μm images were recorded from as-prepared coating at room temperature in air. The z-scale is the same for all images. RMS roughness values are averages of 5 micrographs on the same sample.

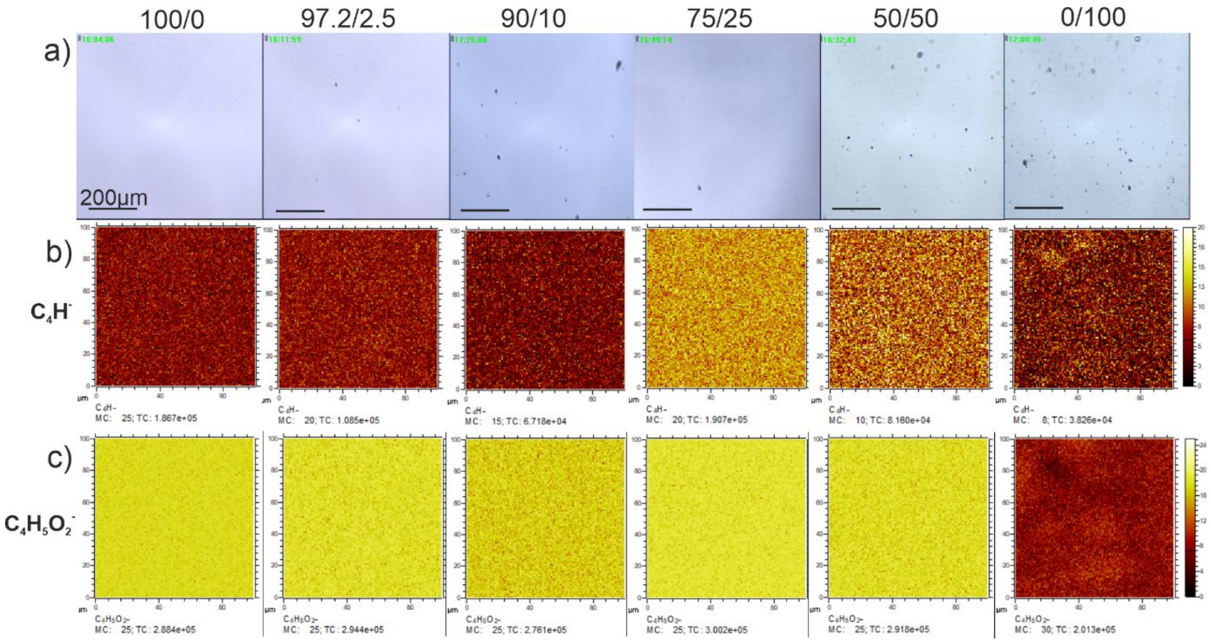

Figure S3. Examination of the P(OEGMa-co-MAA) brush coatings uniformity. (a) Optical microscopy images as well as TOF-SIMS ion maps (100 μm x 100 μm) of (b) C_4H^- and (c)

$C_4H_5O_2^-$ ions characteristic for MAA and OEGMA segments, respectively, recorded for coatings with different composition.

Table S4. Wettability of the copolymer brush coatings. Numbers in brackets are standard errors of the mean from several contact angle measurements of the same sample.

P(OEGMA-co-MAA) composition	water static contact angle
100/0	66.5(1.2)°
97.5/2.5	64.7(0.7)°
90/10	60.7(0.6)°
75/25	56.7(0.8)°
50/50	53.0(1.8)°
0/100	53.2(1.1)°

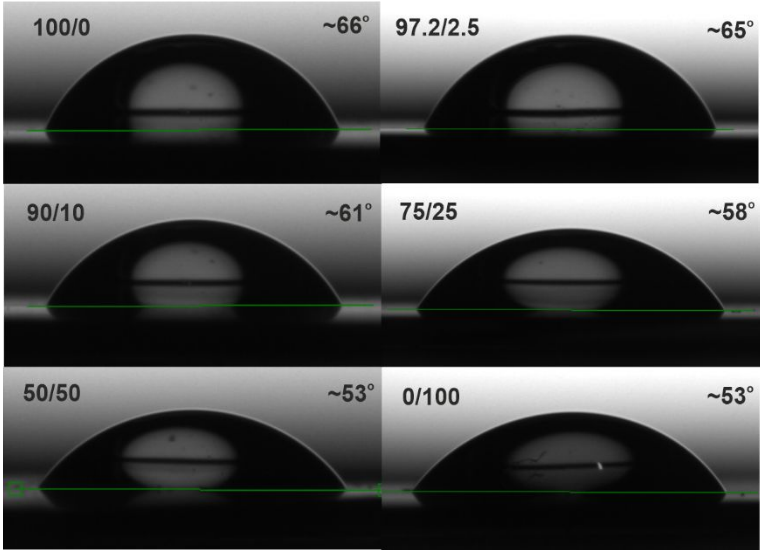

Figure S4. Images of water droplets on the surface of the P(OEGMA-co-MAA) brush coatings with different composition.

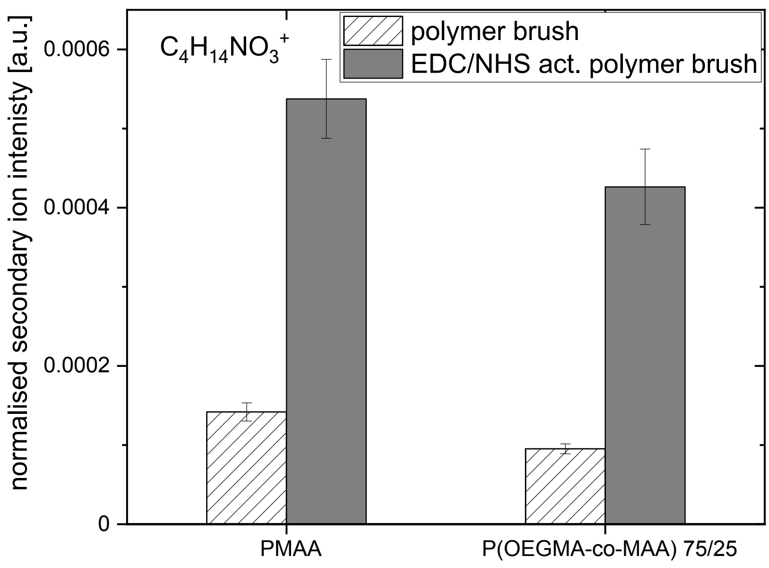

Figure S5. Intensity of the TOF-SIMS signal $C_4H_{14}NO_3^+$, characteristic for the N-hydroxysuccinimide group, recorded for the PMAA and P(OEGMA-co-MAA) 75/25 polymer brush coatings before and after activation of the MAA segments with the EDC/NHS covalent coupling procedure (see Figure 5a,b).

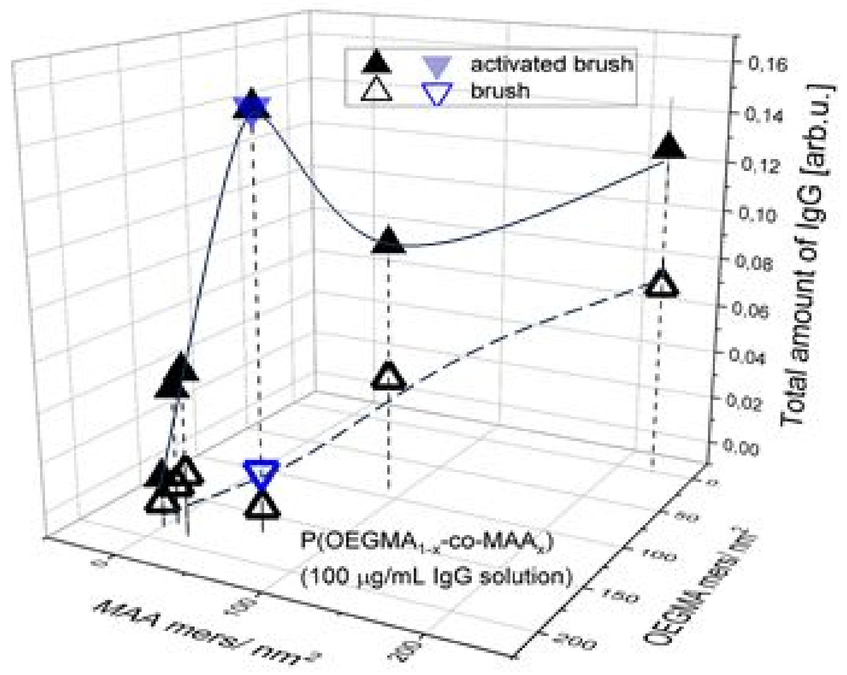

Figure S6. Total amounts of the IgG antibody in the P(OEGMA$_{1-x}$-co-MAA$_x$) brush coatings analyzed as a function of the surface density of both mers, which complement Figure 6a. Copolymer brushes after physical adsorption (open symbols) or covalent immobilization (solid symbols) of IgG antibody, the latter enabled by an earlier brush activation using the EDC/ NHS coupling procedure, were examined.

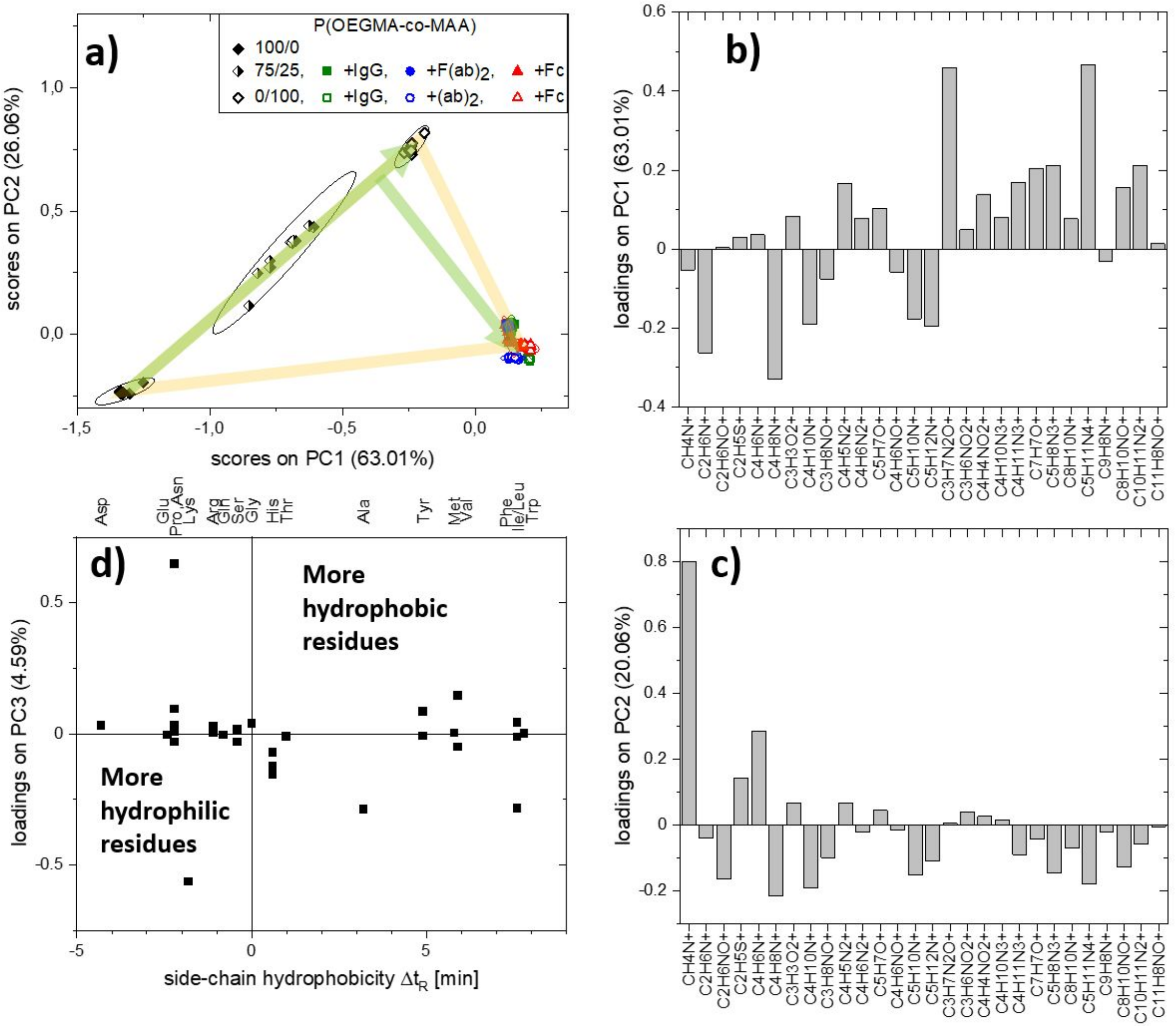

Figure S7. The results of the PCA analysis of ToF-SIMS data, which complement Figure 7c-f, presented for the IgG antibodies attached to the PMAA and P(OEGMA-co-MAA) 75/25 brushes, in addition to the reference layers of the antibody fragments F(ab)$_2$ and Fc and the bare coatings (POEGMA, P(OEGM-co-MAA), PMAA). (a) PC1 vs. PC2 score plot, which reflects

the combined information (marked by arrows) on the composition and the surface coverage of the polymer brush with proteins, and (b, c) the corresponding loading plots for PC2 and P3. (d) Loadings on PC3 from amino acid ion fragments of IgG antibody plotted for each amino acid as a function of its side chain hydrophobicity, defined[a] as the difference in the retention time Δt_R, relative to the Gly peptide, of a peptide analogue differing only by one amino acid residue.

[a]Monera OD, Sereda TJ, Zhou NE, Kay CM, Hodges RS (1995) Relationship of sidechain hydrophobicity and α-helical propensity on the stability of the single-stranded amphipathic α-helix. J Pept Sci 1 (1995) 319–329. https://doi.org/10.1002/psc.310010507
